# A testis-expressing heme peroxidase *HPX12* regulates male fertility in the mosquito *Anopheles stephensi*

**DOI:** 10.1038/s41598-022-06531-x

**Published:** 2022-02-16

**Authors:** Seena Kumari, Sanjay Tevatiya, Jyoti Rani, Tanwee Das De, Charu Chauhan, Punita Sharma, Rajkumar Sah, Shailja Singh, Kailash C. Pandey, Veena Pande, Rajnikant Dixit

**Affiliations:** 1 Laboratory of Host-Parasite Interaction Studies, ICMR-National Institute of Malaria Research, Dwarka, New Delhi, 110077 India; 2 Special Center for Molecular Medicine, Jawaharlal Nehru University, New Delhi, 110067 India; 3 Department of Biotechnology, Kumaun University, Nainital, Uttarakhand India

**Keywords:** Cell biology, Genetics, Molecular biology

## Abstract

In vertebrates dysregulation of the antioxidant defense system has a detrimental impact on male fertility and reproductive physiology. However, in insects, especially mosquitoes the importance of sperm quality has been poorly studied. Since long-term storage of healthy and viable sperm earmarks male reproductive competency, we tested whether the heme peroxidase, a member of antioxidant enzyme family proteins, and abundantly expressed in the testis, also influence male fertility in the mosquito *An. stephensi*. Here, we show that a heme peroxidase 12 (*HPX12*), is an important cellular factor to protect the sperms from oxidative stress, and maintains semen quality in the male mosquito reproductive organ. We demonstrate that knockdown of the *HPX12* not only impairs the sperm parameters such as motility, viability but also causes a significant down-regulation of MAG expressing transcripts such as ASTEI02706, ASTEI00744, ASTEI10266, likely encoding putative Accessory gland proteins. Mating with *HPX12* knockdown male mosquitoes, resulted in ~ 50% reduction in egg-laying, coupled with diminished larval hatchability of a gravid female mosquito. Our data further outlines that increased ROS in the *HPX12* mRNA depleted mosquitoes is the ultimate cause of sperm disabilities both qualitatively as well as quantitatively. Our data provide evidence that testis expressing *AsHPX12* is crucial for maintaining optimal homeostasis for storing and protecting healthy sperms in the male mosquito’s reproductive organs. Since, high reproductive capacity directly influences the mosquito population, manipulating male mosquito reproductive physiology could be an attractive tool to combat vector-borne diseases.

## Introduction

Certain species of mosquitoes are medically important insect pests because they transmit various infectious diseases such as malaria, dengue, chikungunya, and Zika virus. Seasonal variation has a direct impact on the mosquito’s population abundance, and thereby the disease transmission. Although the use of chemical insecticides still holds the key to the success of the vector control program, growing resistance urges the design of new alternative tools^1^. Since the high reproductive capacity of these mosquitoes contributes to their role as disease vectors, a strategy of disrupting sex-specific reproductive physiology could be an attractive tool to combat vector-borne diseases^2–6^. Compared to female mating physiology underlying mating behaviors we have very limited knowledge about their male counterparts.

Mosquitoes have evolved with the complex nature of mating behavior and reproduction^7^. After emergence, male mosquitoesgain sexual maturity in order to be competent for copulation while swarming^8–10^. In nature, it is not only the morphological features such as body size, age and diet which serve as an important indicator for male fitness, but also other factors such as temperature, swarm size and time of swarming have an impact on the reproductive physiology, sperm quality, and seminal fluid diversity^11–14^.

A successful mating event requires an optimal delivery of seminal fluid comprising secretions from the sperm-containing testis, male accessory glands, and seminal vesicle to the female reproductive tract. Though the molecular nature and complexity of this mixture is not fully understood, a series of studies in different insects/mosquitoes have convincingly evidenced that Male accessory gland (MAG) originating seminal fluid, influences the reproductive physiology of the mated female right from sperm deposition in the reproductive tract to egg laying^15,16^.

Increasing evidence highlights that secretions from MAG are essential not only to alter post-mating female behavior, also maintaining various physiological responses such as sperm storage, oogenesis, ovulation, oviposition, and fertility in mosquitoes^17–19^. Mature sperms do not move alone, instead typically accompanied by a molecular trail of Acps, peptides, and steroid hormones, and MAG proteins binding to the sperm tails may also influence the female reproductive physiology^20,21^. Furthermore, a study by Thailayil et al. showed that mating with infertile males induces a similar transcriptional modulation in female reproductive genes as those elicited by fertile males. Such females retained their normal postcopulatory responses, such as refractoriness to remating and oviposition behavior^22^. In recent years, there has been a rapid expansion in our understanding of the male insects’ seminal fluid proteins. However, it is difficult to designate the role of individual components of the male mosquito ejaculate on reproductive success.

Using genomic and proteomic approaches, at least 46 putative *Drosophila* homolog Acps have been identified in the mosquito *An. gambiae*, but their functional correlation has not been fully established^23^. Knockdown of *An. gambiae* ortholog of Acps a regulating heat shock factor (HSF) not only reduces the expression of Acp transcripts such as Acp62F*(AGAP006587)*, Acp70A (*AGAP009352*), and Acp26Aa but also affects the oviposition/hatching rate, when mated with wild-type female *An. gambiae*^24,25^. Earlier studies also highlights that male-transferred various horomones such as 20-hydroxyecdysone (20E) and juvinile hormone (JH-III) influences several aspects of female mosquitoes behavior^26,27^, but their role in the regulation of male fertility is obscure.

Recently, a study by *Peirce *et al.^28^ showed that 20E-triggered oviposition is regulated by the stress and immune-responsive JNK signaling pathways. The heads of mated females exhibit a transcriptional signature reminiscent of a JNK-dependent wounding response. This study proposes an unusual linking of stress responses and reproductive success in *An. gambiae*^29^. Studies highlight that an uncontrolled production of reactive oxygen species (ROS) may affect spermatozoa activity, damage DNA structure, and accelerate apoptosis, sperm motility, viability, and numbers^30–32^. Previously, a mating-induced heme peroxidase (HPX15), an enzyme that plays an important role in the maintenance of optimal reactive oxygen species (ROS) level, has also been implicated as an important factor for long term-storage of healthy sperms in the spermatheca of mated adult female *An. gambiae*^33^. However, despite the important contribution of male mosquitoes in the mating success and alteration of vector population dynamics^34^, so far similar mechanism that how it facilitates healthy sperm storage and influences male fertility remains elusive.

*Anopheles stephensi* is a major malarial vector that transmits about 20% of urban malaria in India. With the increased challenge of using IRS (Indoor residual spray)/LLINs (long lasting insecticidal nets) due to insecticide resistance, larval control is recommended in urban settings^35^, but it does not suffice to control adult mosquitoes. Unlike *An. gambiae*, the reproductive physiology of *An. stephensi* has been poorly investigated, except for the classical assessment of age and mating status on male reproductive fitness^36^. Here, we unravel a functional correlation of testis expressing *As-HPX12* with the maintenance of male fertility and reproductive physiology in mosquito *An. stephensi*.

## Material and methods

Supplemental Fig. 1 represents an overview of the technical design and experimental workflow.

### Basic entomology

#### Mosquito resaring

*An. stephensi* mosquitoes were reared under standard conditions (26 °C–28 °C, 70–80% relative humidity, 12:12 day:light)^37,38^. Eggs were floated in a pan filled with deionized water and once larvae hatched, approximately 1000 larvae were reared in an iron tray (66 cm × 45 cm × 17 cm). Larvae were fed on a 1:1 mixture of dog food (Pet Lover’s crunch milk biscuit, India) and fish food (Gold Tokyo, India). Post-emergence adult mosquitoes were fed daily on sterile sugar solution (10%) using a cotton swab, and for routine oviposition and gonotrophic cycle maintenance, blood was offered from a rabbit. All protocols for rearing and maintenance of the mosquito culture were approved by the ethical committee of the National Institute of Malaria Research, New Delhi (NIMR/IAEC/2017-1/07; dated 28/09/2017).

#### Pupae sexing and smosquito mating

Pupae were sexed individually using a light microscope, and placed in dishes filled with deionized water in separate cages (40 × 30 × 40 cm) to maintain virginity. Immediately after emergence, cages were also inspected to ensure proper sorting of male and female mosquitoes. Mating assays were performed overnight, where 100 virgin females, 3–4 days after eclosion, were kept in a cage with equal number of age-matched male mosquitos. Mating success was assessed by dissecting and visualizing live sperm in spermathecae of randomly selected 8–10 mosquitoes (Supplemental Fig. 6), in addition to the verification through sperm-specific primers ams (*ASTEI07690*) and mts (*ASTEI04832*) by RT-PCR in virgin *vs.* mated spermatheca, as described earlier^39,40^.

### Molecular biology

#### RNA extraction, cDNA preparation, and quantitative RT-PCR

Experimentally required tissues were dissected and pooled from the cold anesthetized adult female and male mosquitoes under different conditions *i.e.* virgin and mated. To examine the tissue-specific expression of target genes, selected tissues such as hemocyte^38^, spermatheca, salivary gland, male reproductive organ (male accessory gland, testis) were dissected from age-matched ‘control’ *vs*. ‘test’ mosquitoes. To evaluate the abundancy of *HPX12* expression further we dissected and separated both sub-tissues (MAG and testis) from the same male reproductive organ (MRO) of virgin male mosquitoes (30 numbers). Total RNA from collected tissues was isolated using the standard Trizol method as described previously^41^. RNA was quantified by using a Nano-Drop Spectrophotometer (Thermo Scientific). Isolated ~ 1 µg total RNA was utilized for the synthesis of the first-strand cDNA using a mixture of oligo-dT and random hexamer primers and Superscript II reverse transcriptase as per the described protocol (Verso cDNA synthesis Kit, Cat#AB-1453/A, EU, Lithuania). For differential gene expression analysis, routine RT-PCR using gene specific primers (see Supplemental Table 1), and agarose gel electrophoresis protocols were. The relative abundance was assessed using the SYBR Green qPCR master mix (Thermo Scientific), by CFX96 PCR machine (Bio-Rad, USA). PCR cycle parameters involved an initial denaturation for15 min at 95 °C, 40 cycles of 10 s at 95 °C, 15 s at 52 °C, and 22 s at 72 °C. After the final extension step, melting curves were derived. Each experiment was performed in three independent biological replicates. The relative quantification results were normalized with an internal control (Actin), analyzed by the 2^−ΔΔ^Ct method.

#### Gene knockdown assays in adult mosquitoes

To knockdown or silence the *AsHPX12* mRNA expression, dsRNA primers carrying T7 overhang were synthesized as listed in the Supplemental Table 1, following the protocols as described earlier^42,43^. The amplified PCR product was examined by agarose gel electrophoresis, purified (Thermo Scientific Gene JET PCR Purification Kit #K0701), quantified, and subjected to double-stranded RNA synthesis using Transcript Aid T7 high-yield transcription kit (Cat# K044, Ambion, USA). The bacterial dsr*LacZ* gene was used as a control^42,43^. Approximately ~ 69 nl (3 µg/µl) of purified *dsRNA* product was injected into the thorax of a cold anesthetized 1–2-day old male mosquito using a nano-injector (Drummond Scientific, CA, USA), as described earlier^44,45^. The knockdown of the respective gene was confirmed by quantitative RT-PCR after 3–4-days of dsRNA injection.

### Microscopic assay

#### MRO morphological examination

To track the possible role of *HPX12* in male reproductive organ morphology, initially we design basic microscopic assays where live males were cold anesthetized, placed in a drop of phosphate-buffered saline (PBS) over a microscopic glass slide, and examined under a dissecting microscope (16 ×) (Supplemental Fig. 2). Their reproductive system was removed using minutes needles to pull out the last segment of their abdomen. Slow excision removed the whole male reproductive system, including the testis, accessory glands, and ejaculatory duct, and subsequent observations were made under a compound microscope (10 ×/40 ×/100 × immersion oil). To visualize and capture images of the target tissue/organ male reproductive organs, including the testis and accessory glands, we used the microscope-assisted digital camera–computer system (BM-X LMI). The raw images of both the control and test groups, captured in identical magnifying parameters, were processed either using Image J 1.240 software, or professional software (Microsoft Photo Edit software; version 2020.19111.24110.0).

#### Sperm motility and quantification

On the microscopic slide the fresh dissected male reproductive organ, was covered with a coverslip, and mechanically pressed with very gentle pressure enough for organ rupture and release of live sperms into the solution (110 mM NaCl, 5 mM KCl, 0.5 mM CaCl_2_, 1.2 mM MgCl_2_, 1.2 mM MgSO_4_, 1.2 mM Na HCO_3_, 2 mM KH_2_PO_4_, 2 mM Na_2_ HPO_4_, 1 mM glucose, 20 mM HEPES, pH 7.4) at room temperature. The sperms were examined using 40 × objective lens (400 × magnification) under the microscope, and the raw video was captured in the computer through a digital camera (BM-X LMI). Motility of the sperms and their associated fluid movement was recorded in the age-matched two groups i.e. control *vs* silenced mosquitoes within the same time frame under the camera/video option and analyzed through ImageJ 1.240 software (National Institutes of Health, Federal Government of the United States; Supplemental Fig. 2). For quantitative analysis, we used Trackmate software, an open-source Fiji plugin for the automated as well as manual tracking of single-particle^46^. We randomly selected 30 sperms to analyse the motility of sperm from each group of control and silenced. Images of time-lapse series were converted to .tif files and saved in one folder per series. Then, we imported the images to Fiji via Plugins from one time-lapse series as one hyper stack using the BioFormats import function. Under the selected TrackMate tool option available in the Fiji toolbar, the sperm to be tracked was targeted as a green circle with dashed lines after a double click on it. After setting the size and position of the one tracker which turns from the dashed green lines into a solid green line, we set out the next frame in the time-lapse series. To set the new location of the tracked sperm in the new frame, hovering the mouse over the new point, where the tracker appears to the new location. A line connects the locations where the tracker has been placed in the previous frames. Following the completion of the traces, the data was generated through the *Analyze* option in the TrackMate dialogue box and calculated the speed by dividing the total path length of the sperm by the elapsed time.

#### Sperm viability assay

We separated complete MRO (testis, seminal vesicles, and MAG) from matured virgin male mosquitoes in spermatozoa assay buffer (145 mM NaCl,4 mM KCl,1 mM MgCl_2_,1.3 mM CaCl_2_,5 mM d-glucose and 10 mM 4-(2 hydroxyethyl)-1-piperazine ethane-sulfonic acid) pH 7.4,10% DMSO), and collected in the 1.5 µl Eppendorf tube, containing 100 µl assay buffer for gentle mixing by smooth and very slow movement (up/down) of pipetting. The sperm cells were stained with 1 µl SYBR 14 (1:50 in DMSO) and 5 µl propine iodide (PI) dye (ThermoFisher #L7011) and incubated for 2–3 min^47^. ~ 10ul of the sample was loaded on a microscopic slide and examined at 400 × magnification under phase contrast, and fluorescence microscopy (BX61 Olympus) employing a mercury excitation beam at 480/20 and 535/30 nm filters to assess green and red fluorescence, respectively. Two images, one for each fluorescence filter, were captured from each field and processed using cellSens software.

#### ROS determination assay

To determine the level of ROS generation, MRO (MAG, Testis and seminal vesicles) from *HPX12* silenced or control mosquitoes were collected in PBS and incubated with a 2 mM solution of the oxidant-sensitive fluorophores, CM-H2DCFDA [5-(and-6)-chloromethyl-29,79-dichloro-dichlorofluorescein diacetate, acetyl ester] (Sigma) for 20 min on a microscopic glass slide. After a series of experimental trial, the final concentration of DCFDA was optimized to 0.2 mM^41^. Following, incubation for 20-min at room temperature in the dark, the MRO was washed three times with 1xPBS. Next, the MRO was transferred to a new glass slide in a drop of fresh PBS and checked the fluorescence at 400 × magnification under a fluorescence microscope, which emits a mercury excitation beam to assess green fluorescence at 480 nm and 530 nm, respectively. Fluorescent images were acquired using an inverted fluorescent microscope (BX61 Olympus), with a CC12 fluorescence camera (Olympus Co., Tokyo, Japan). Pixel intensity within microphotographs was determined and analyzed using Image J 1.240 software, and the redox state measurements were expressed as arbitrary units.

#### Morphological assay in spermatheca

4–6 day old adult female mosquitoes were allowed overnight to mate with males in a 1:1 ratio. Post mating, males were removed and females were secured back to insectary. After 24 h of mating, each female’s spermatheca was dissected in 20 μl fresh saline on a microscopic slide. Using a coverslip the dissected spermatheca was gently placed, and the uncracked spermathecae were observed under a compound microscope. To visualize the sperms, spermatheca was gently cracked with mild pressure on the coverslip by slowly wicking saline with a needle, and performed the microscopic analysis.

#### Oviposition assay

Overnight mated 50 females (mated either with control or *HPX12* knockdown male mosquitoes) were blood-fed over a rabbit. After blood-feeding, partial/incomplete blood-fed females were removed, while fully engorged females were kept in a cage for 48 h, and then 30 females individually placed in netted plastic cups (10 cm diameter × 5.75 cm depth), containing 1/3rd-filled water, and fixed with blotting paper on which to lay eggs. After 24 h the total number of eggs laid on blotting paper were manually counted and compared among both mosquito groups. The number of eggs laid, and hatched larvae (within 72 h) were recorded in three independent experiments.

### Statistical analysis

Statistical analysis was performed using Origin8.1 software. Initially, all the relative expression data were evaluated for a general response through multiple comparison using one way analysis of variance (ANOVA), however, wherever required “test” sample data was compared with “control” data set and statistically analyzed using Student’s t-test. All the data were expressed as mean ± SD, and final *p-*values were adjusted using the Benjamini & Hochberg test. The *Mann*–*Whitney U* test was used to evaluate and analyze egg-laying and hatching experiments, where results were considered significant if the *p*-value was less than 0.05 Each experiment was performed at least thrice to validate the findings. Quantitative analysis of sperm motility/viability/fluorescent intensity was statistically calculated using Student’s *t*-test, where *p* < 0.05 was taken as significant.

### Animal ethics approval statement

All necessary methods for the use of Live Rabbit were duly approved by Institute Animal Ethics Committee (NIMR/IAEC/2017-1/07; dated 28/09/2017); and carried out in accordance with relevant ARRIVE guidelines and regulations.

### Consent for publication

All authors agreed to this publication.

## Results

### Mating alters *HPX12* gene expression in male mosquito reproductive organs

**Figure 1:**
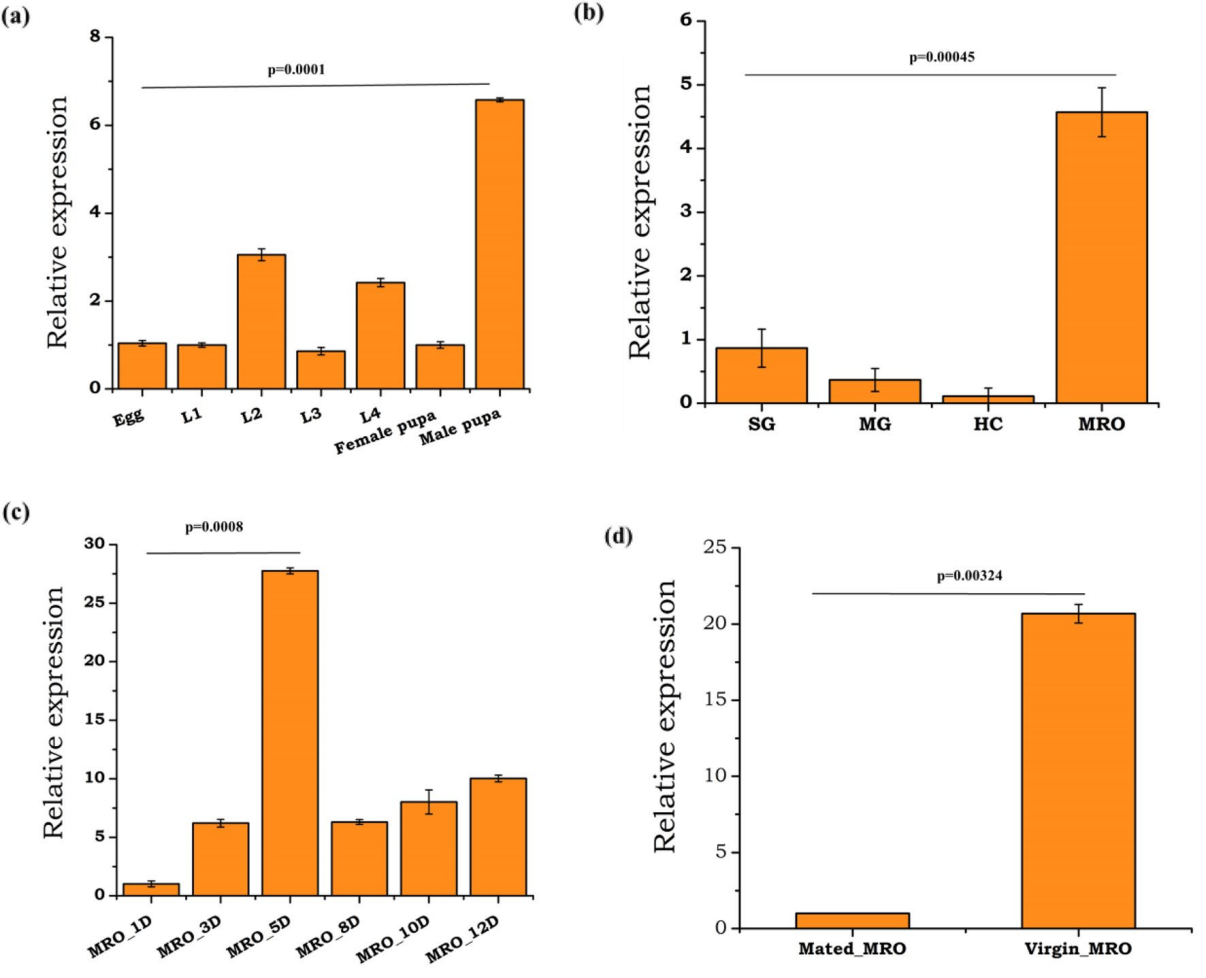
Transcriptional profiling of the heme peroxidase 12 (*HPX12*) in mosquito *An. stephensi* (**a**) *HPX12* expression during embryonic development: L1—larval instar one, L2—larval instar two, L3—larval instar three and L4—larval instar four, pupa (*HPX12*/p < 0.0001) (n = 10, N3), Egg sample was considered as a control for each test sample; (**b**) Tissues specific expression kinetics of *HPX12* in male mosquito tissues. *SG* Salivary glands, *MG* Midgut, *HC* Hemocytes, *MRO* male reproductive organs (*HPX12*/p < 0.00045), where male salivary glands were considered as control for each test sample; (**c**) Age-dependent expression of *HPX12* in virgin male mosquito reproductive organ (MRO) i.e. 1D(day),3D,5D (*HPX12*/p < 0.0008), 8D and 10D, 12 days, where male (one-day-old) mosquitoes were considered as a control for each test sample. (**d**) Mating-induced changes in *HPX12* in male reproductive organ Virgin vs. Mated (*HPX12*/p < 0.00324). Final *p*-values were adjusted using Benjamini & Hochberg test; Three independent biological replicates (n = 30, N = 3) were considered for statistical significance *p < 0.05; **p < 0.005 and ***p < 0.0005 was calculated using paired Student’s *t*-test. (*n* = represents the number of mosquito pooled for sample collection; *N* = number of replicates).

Because of a very high rate of cell division and mitochondrial oxygen consumption in testicular tissue, the onset of the spermatogenesis process is highly susceptible to oxidative stress during the early development of male gonads^48,49^. Initially, we evaluated the expression pattern of at least 10 transcripts including six heme-peroxidase (HPX) family members (Supplemental Fig. 3a), and four non-HPX members such as catalase, superoxide dismutase (SOD1, SOD2), and Glutathione-S-Transferase (GST) (Supplemental Fig. 4c), during the aquatic development of the mosquito. Although we noticed that all the tested genes showed altered expression throughout the development, the expression of HPX12 was exceptionally enriched in the pupae as compared to other stages (Fig. 1a; also see Supplemental Figs. 3a, 4c). A sex-specific relative expression analysis further showed elevated expression of *HPX12* in the male pupae than females (Supplemental Fig. 4a,b). Similarly, a higher expression of antioxidant stress (AOS) related transcripts was observed in newly emerged males than in female mosquitoes (Supplemental Fig. 4a,c), where *HPX12* expression level was > fourfold (p < 0.00045) higher in the male reproductive organ (MRO) than midgut, salivary glands, and hemocytes (Fig. 1b). Next, we observed an unusual pattern coinciding with enriched expression of all HPX members (Supplemental Fig. 3c), including HPX12 (Fig. 1c), during young (3-5Days) or aged (12D) mosquitoes. Surprisingly, the expression of *HPX12* remains exceptionally higher than other tested heme peroxidase transcripts in unmated virgin male MRO (Supplementary Fig. 3d). After 24 h of mixing , a significant loss was also observed in the expression level of *HPX12* (p < 0.0003) (Fig. 1d), allowing us to hypothesize and test its possible role in the male fertility.

### HPX12 knockdown influences sperm functionality in *An. stephensi*

**Figure 2:**
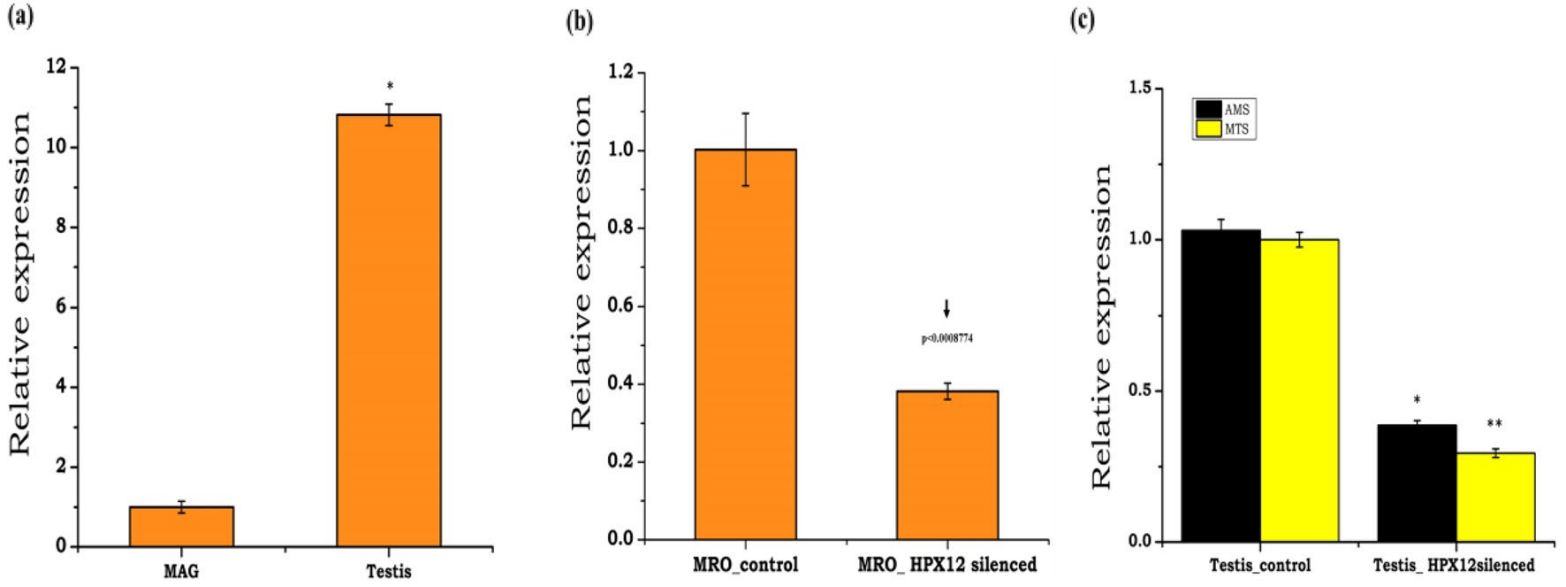
HPX12 and sperm-specific transcripts profiling in male reproductive organs: (**a**) HPX12 relative expression in MRO i.e. MAG: male accessory gland and testis (p < 0.01504); (**b**) *HPX12* knockdown exhibited a ~ 80% reduction in mRNA level as compared to control mosquito (p < 0.0008774); (**c**) Co-expression of sperm-specific genes ams (p < 0.0067), mts (p < 0.001) in the control and *HPX12* silenced mosquitoes. Three independent biological replicates (n = 30, N3) were considered for statistical significance *p < 0.05; **p < 0.005 and ***p < 0.0005 was calculated using unpaired Student’s *t*-test. (*n* = represents the number of mosquitoes pooled for sample collection; *N* = number of replicates).

To test and establish whether *HPX12* influences sperm functionality, initially, we compared the relative expression of *HPX12* in the MAG and testis. We noticed a > tenfold higher expression of *HPX12* (p < 0.05; Fig. 2a) in the testis, as well as co-expression of sperm-specific markers, namely ams/mts, in the testis of aging adult male mosquitoes (Supplemental Fig. 5a,b). Surprisingly, a perfect alignment with *HPX12* expression pattern in both mated and unmated mosquito (Supplementary Fig. 5c), and a relative loss in the expression of ams (p < 0.0067) and mts (p < 0.001) (Fig. 2c) after knockdown of *HPX12* (> 80% downregulation; (p < 0.00087/Fig. 2b), together with suggested a potential role in the optimal maintenance of sperm storage and survival in the male reproductive organ. To further test and evaluate a possible impact of *HPX12* knockdown on sperm functionality, we examined key parameters such as sperm viability, motility defining the quality of sperm in the male mosquitoes.

**Figure 3:**
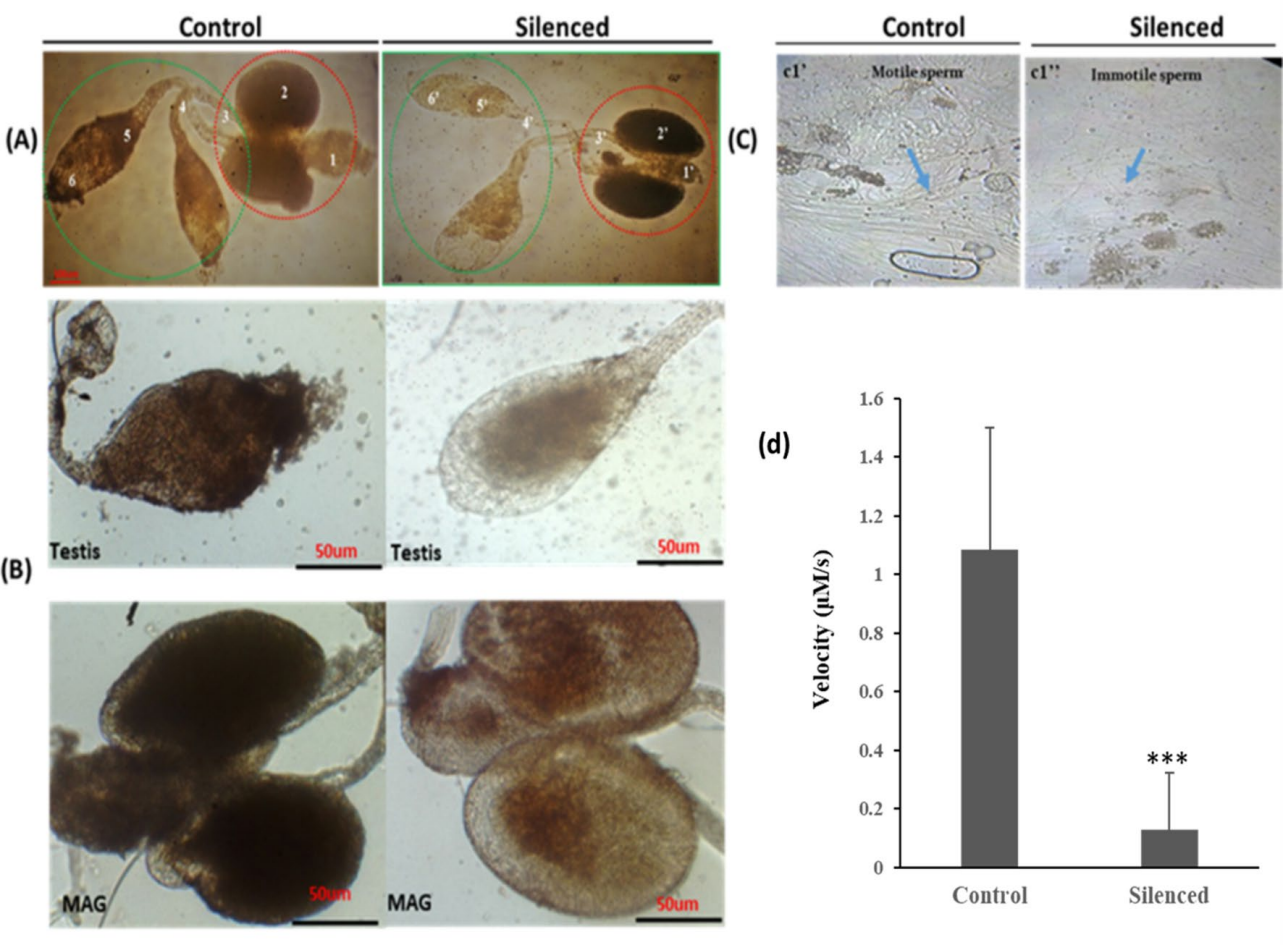
*HPX12* knockdown affects male reproductive organ (MRO) integrity and sperm physiology: The reproductive system of control and silenced male mosquitoes were dissected and placed in a drop of phosphate-buffered saline (PBS) and examined under a dissecting microscope (16 ×). (**A**, **B**) Phase contrast microscopy and comparative morphological overview of the complete MRO features i.e. numbers on the figure represented as a’ 1—ejaculatory duct, 2—accessory glands, 3—seminal vesicle, 4—vas efferentia 5—sperm reservoir, and 6—spermatocytes; Knockdown of *HPX12* cuases the loss in the cellular integrity of the testis (green circle/also see panel B upper image) as well as MAG (red circle/also see B lower image); (**C**) Effect of *HPX12* knockdown on sperm quality (motility), examined under a light microscope (40 ×), gently ruptured male testis/seminal vesicles from control (c1’) and *HPX12* knockdown (c1”) mosquito group, were visualized in PBS, highlighting motile (curved shaped) healthy and abnormal sperm structure (light blue arrow), respectively (also see Videos S1–S6); (**D**) *HPX12* knockdown reduces the motility of the sperm (p < 0.0005); quantitative analysis was carried out through automated Trackmate software by importing time-lapse series images, caputured from live videos, and labelled with colored tracker indicator for target sperms. Compared the motility as distance/time for both control and knockdown male mosquitoes’ sperms, and statistically analyzed using Student’s *t*-test-(p < 0.0017).

Our initial phase-contrast microscopy and video capture analysis indicated a significant loss in the integrity of the male reproductive organ (Fig. 3A,B; Supplemental Video S2–S4: video clips). Although, the mechanism of this alteration is yet to be unraveled, however, increased stickiness to the needles was readily evident while dissecting and removing the male reproductive organ (MRO) from *HPX12* knock down mosquitoes. Additionally, we also observed a significant loss in the motility of the sperms in the HPX12 knockdown mosquitoes (Fig. 3C,D). A quick assessment of an increased number of dead sperms, by trypan blue staining which marks the sperm head of the dead cell^50^, suggested that disruption of *HPX12* may have an adverse impact on sperm qualities such as loss in motility, viability, and testicular physiology (Supplemental Fig. 2).

**Figure 4:**
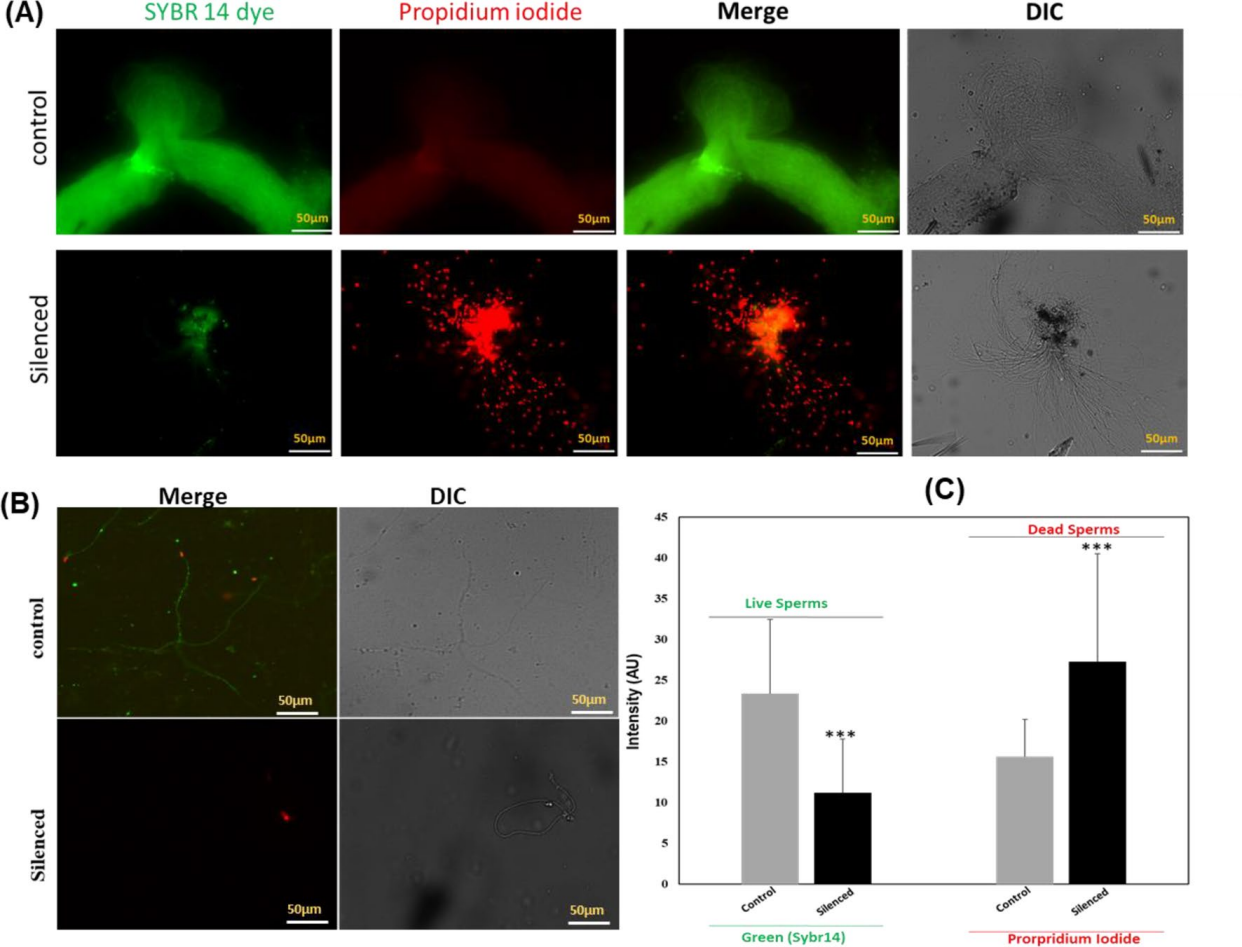
*HPX12* regulates sperm quality: (**A**, **B**) Fluorescent microscopy analysis of mosquito sperm viability: Control mosquito sperms appear green due to uptake of SYBR14 only; while *HPX12* knockdown male mosquito sperm are red due to uptake of PI. Overlaid fluorescence images of control and *HPX12* knockdown MRO represent dual staining with SYBR-14 and propidium iodide to distinguish green-fluorescing live from red-fluorescing dead spermatozoa; *DIC* differential interference contrast. (**C**) Quantitative analysis of fluorescent intensity, for which the images were processed in an isogenic graphics environment using ImageJ software, and the data was statistically analyzed using a Student’s *t*-test. Standard error bars are shown and stars indicate p < 0.0005; three independent biological replicates (n = 15, N = 3) were considered for statistical significance (*n* = represents the number of male msoquitoes dissected to pool MRO for sample collection; *N* = number of replicates).

Thus to further evaluate and quantify the effect of sperm qualities, we performed a viability assay that utilizes differential staining methods i.e., SYBR14 (green) for live and Propidium Iodide (red) for dead sperms. The increased green intensity in naïve (control), and red in the knockdown mosquito group (Fig. 4), together confirmed and validated that *HPX12* have an important role in the maintenance of healthy and viable sperm possibly by regulating reactive oxygen species (ROS) level^51,52^.

**Figure 5:**
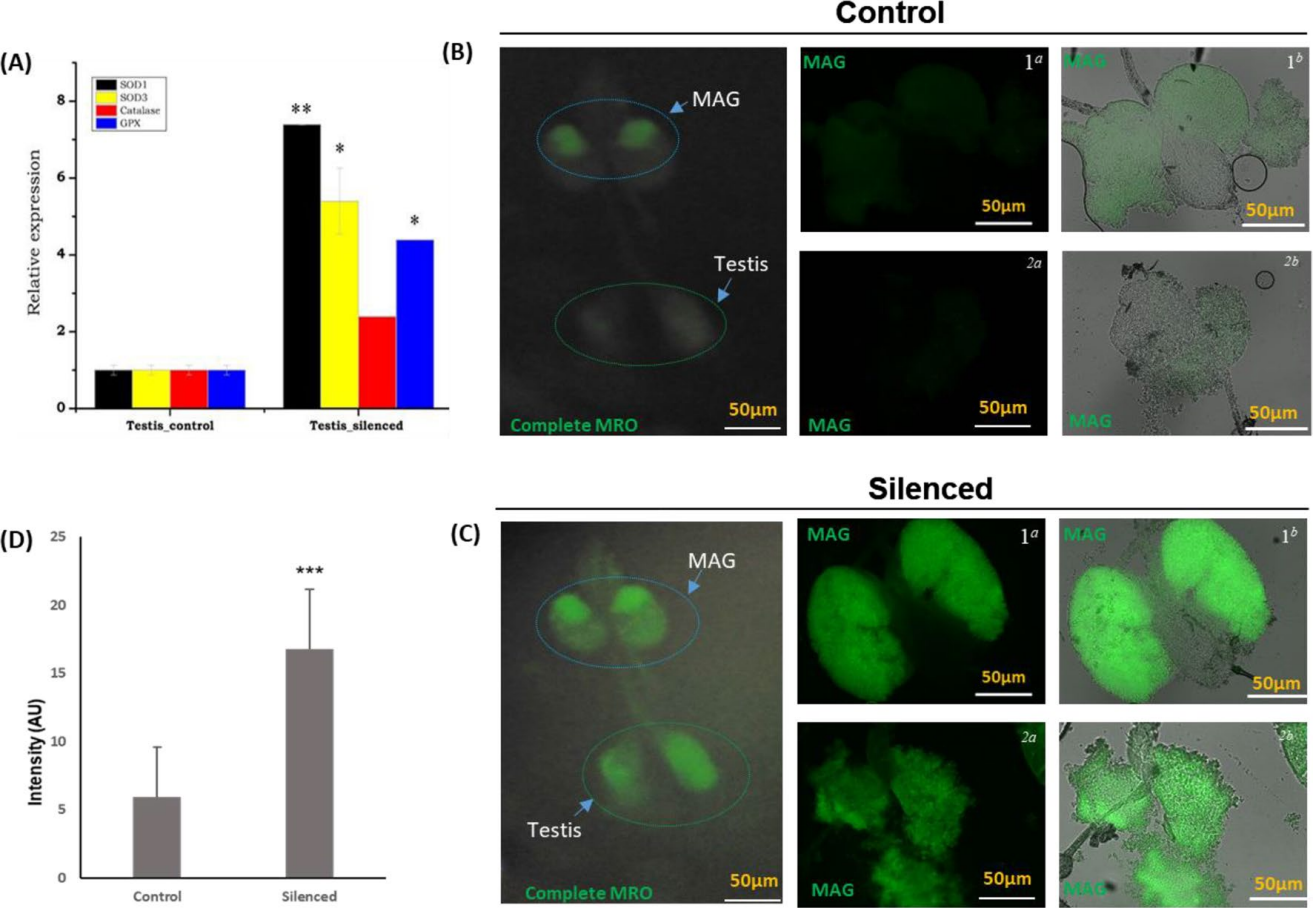
*HPX12* knockdown impairs anti-oxidative defense system: (**A**) Relative antioxidant enzyme transcripts expression in response to increased oxidative stress after *HPX12* knockdown: SOD1 (p < 0.00072), SOD3(p < 0.042), and GPX (p < 0.0085); (**B**, **C**) Comparative fluorescent microscopic analysis demonstrating the effect of *HPX12* knockdown on ROS generation in the male reproductive organ: Control (ds*LacZ*), and silenced (ds*HPX12*) injected virgin male mosquito’s MRO were incubated with DCFDA, a dye leading to increased fluorescence intensity in response to ROS. To ensure *HPX12* impact on the MAG at least 3–4 independent replicates were captured under identical conditions i.e. dark/bright field e.g.1^a^/1^b^ & 2^a^/2^b^, respectively, and (**D**) to calculate the changes in the green fluorescent intensity, the images were processed in an isogenic graphics environment using ImageJ software, and the data were statistically analyzed using a un paired Student’s *t-*test (p < 0.01).

To strengthen the above hypothesis, and trace the possible cause of the sperm qualitative and quantitative loss, first we evaluated the expression of ROS generating transcripts in the *HPX12* knockdown male mosquitoes. We observed a significant up-regulation of all the selected ROS-generating transcripts, notably superoxide dismutase (SOD1/p < 0.0097; SOD3 (p < 0.042), and GPX (p < 0.0085) (Fig. 5a), in the *HPX12* knockdown mosquito group. To further assess the cellular impact of HPX12 knowdown, we performed a DCFDA assay. As expected, our initial observation indicated that HPX12 knockdown affects the whole male reproductive organ (Fig. 5B–D).To ensure the impact on male accessory glands, we also compared the change in the fluorescent intensity of the MAG samples independently (Fig. 5B–D). Together, these observations confirmed that the altered ROS level may have a detrimental impact on the male reproductive organ of the unmated male mosquitoes.

### *HPX12* influences accessory gland proteins (Acps) expression and male fertility

**Figure 6:**
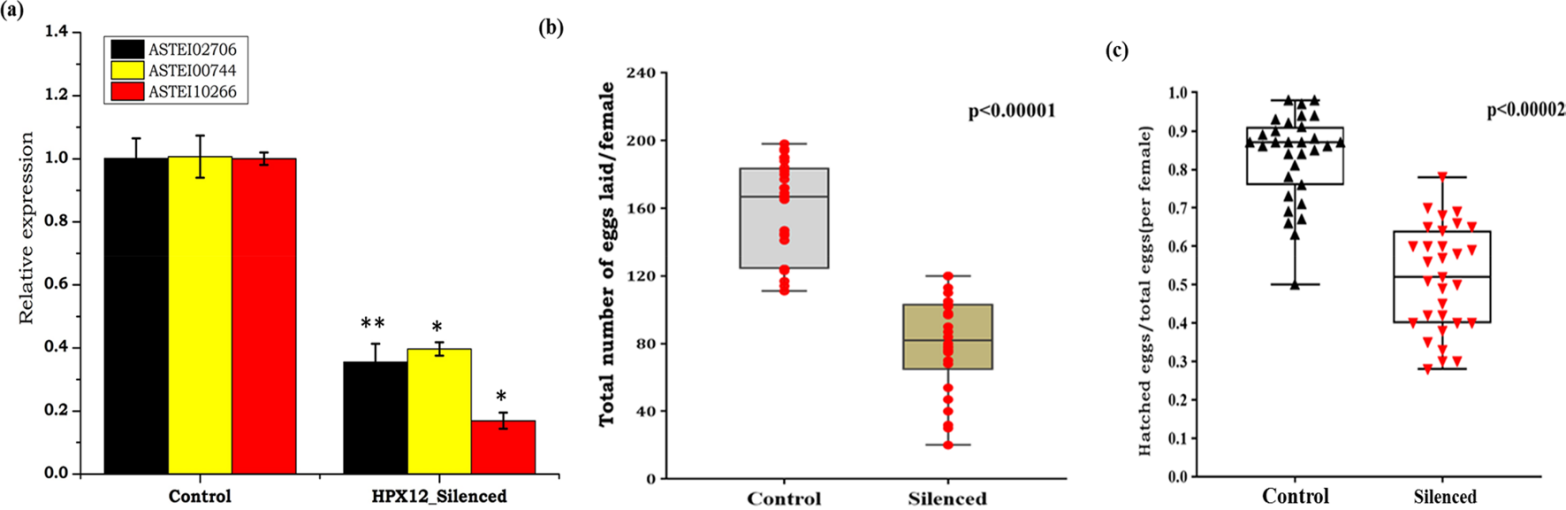
Effect of *HPX12* knockdown on putative Acps encoding transcripts expression (**a**) HPX12 (p < 0.058), *ASTEI02706*(p < 0.003068), *ASTEI00744*(p < 0.0188012) and *ASTEI10266* (p < 0.0375),) depletion in MAG; and reproductive phenotypes i.e. oviposition and hatching (**b**, **c**); in male reproductive organ: Box plot graphs show (**b**) the number of eggs laid by individual females mated with *dsHPX12*‐knockdown males. *Mann‐Whitney U test* with *P* < 0.05 confirmed the statistical significance of the reduction, with *p* < 0.00001; (**c**) number of hatched eggs/total eggs laid from individual females mated with *HPX12*‐knockdown males, with *p* < 0.00002.

Seminal fluid in mosquitoes contains diverse nature of Acps which are key modulators of female sexual behavior and physiologies such as re-mating, longevity, and egg production. Unpredictable variability of acessory proteins (Acps), and limited data on *An. stephensi* mating biology, first we performed a homology search of at least 5 putative transcripts pre-identified from other mosquito species such as *An. gambiae*^23^, against *An. stephensi* database (Supplemenatl Table 2). Common domain structure, acceptable homology (> 30%) and spatio/temporal expression data allowed us to select at least three putative Acps encoding transcripts namely *ASTEI02706*, *ASTEI10266* and *ASTEI00744*, having enriched expression in the MAG of virgin male mosquitoes (Supplemntal Data S8). Next, we also tested whether *HPX12* has any functional correlation with Acps gene expression. Surprisingly, observation of a significant down-regulation of tested Acps transcripts after *HPX12* mRNA silencing in the virgin males, further correlates that *HPX12* may influence Acps, possibly to preserve healthy sperms (Fig. 6a–c). Additionally, our molecular profiling with *ams/mts,* and comparative morphological analysis further indicated that mated female mosquito spermatheca also carries a relatively low sperm load than the control mosquitoes group (Supplemental Fig. 6).

Finally, we tested whether this modulation in Acps expression and low-quality sperm had any effect on oviposition and fertility rates, in the female mosquitoes. When mated with *HPX12* knockdown male mosquitoes, we observed that the number of eggs deposited by each gravid female mosquito was lower than that of the control group in terms of both median 78.16 *vs.* 159.5 as well as mean: 80 *vs* 167, with an average percentage reduction of 50% median and 52.09% for the mean (Fig. 6b, Supplemental Fig. 7). Likewise, we also observed a reduction of the hatched larva number in terms of both median values of 0.87 *vs* 0.52and mean values of 0.83 *vs* 0.516, with an average percentage reduction of 40% median and 37% for the mean (Fig. 6c).

## Discussion

Sperm quality plays an important role in vertebrate species, however, in insects, especially mosquitoes, the importance of sperm quality has been poorly studied, despite sperm competition being widespread and well established^53^. During the insemination process, along with sperms, the male mosquitoes also deliver seminal fluid comprising several proteins, hormones, and other factors to females that inhibit re-mating, alter host-seeking behaviors and stimulate oviposition. Through modern ‘*omic*’ technologies such as bioinformatics, genomics, transcriptomics, and proteomics, attempts are on the way (i) to resolve the molecular composition and complexity of male ejaculates, and (ii) to know how they mediate their effects on female mosquitoes. Appreciably, a comprehensive literature review highlights the important role of MAG originating seminal fluids in the modulation of female mosquito sexual behavioral physiology, however, the equivalent role of sperm quality in unmated adult males, is yet unknown.

Our findings highlight the transcriptional modulation of anti-oxidative stress-responsive proteins after mating and explore the possible role of heme peroxidase in maintaining male mosquito’s fertility. Through comprehensive functional knockdown experiments, coupled with cellular assays and detailed microscopic analysis, here we identified *HPX12*, abundantly expressing in the male testis, and shows that this gene product may likely play an important role in the regulation and maintenance of viable and healthy sperms in the mosquito *An. stephensi*.

Previous studies have suggested that the maturation of sperm mother cells i.e. spermatogonia, coincides with the early development of the gonad system of the male mosquitoes^54^. Our observation of an enriched anti-oxidant system gene expression further supports the idea of spermatogenesis undergoing in the maturing gonads during the early pupal stage^55,56^. However, newly emerged male mosquitoes may remain unfit for sexual courtship until it achieves a morphological change in the external genitalia through inversion of the terminalia within the first 24 h of emergence^57^. In many Anopheline mosquitoes species, male accessory glands develop during the first few days and usually attains maturity at 5–7 days of adult life^56,58^. Mahmood and Resien^56^ showed that after sexual maturity at 3 days of age the male accessory glands are replete with MAG secretions*,* and remain active throughout life in the mosquito *An. stephensi*. They also observed that morphological changes in the male reproductive system closely mirror the changes in mating activity. Further, multiple mating experiments suggested that adult male mosquito's mating frequency peaks between 3 and 7 days of age when the reproductive system became morphologically most competent^56,58^. Corroborating these observations, an age-dependent AOS gene expression data analysis, and their altered expression after mating together confer that laboratory-reared adult male *An. stephensi* may achieve sexual maturity within 3–5 days after emergence, where an active anti-oxidative defense system is necessary to maintain healthy sperm until mating event completion.

An elevated level of *HPX12* expression than other members prompted us to test its potential role in male mosquito’s reproductive physiology and fertility maintenance in *An. stephensi*. We noticed that an effective knockdown of *HPX12* (> 80% reduced mRNA level) not only causes a quantitative loss in the sperms, but also alters the qualitative nature of sperm such as motility and viability. Earlier studies suggest that until mixed with seminal fluid from male accessory glands during ejaculation, sperms remain weakly motile in the dissected male mosquito’s seminal vesicle^59^. However, upon mixing with semen from accessory glands, the sperm progresses sequentially from A- to B- to C-type motility *i.e.* a long wavelength, with low-amplitude flagellar wave (type A); a double-wave pattern of a short-wavelength superimposed over high-amplitude wave (Type B); and a fast helical wave (type C) in the mosquito *Culex quinquefasciatus*^60–62^.

Since in our motility assay we compared the whole male reproductive system (MAG and testis) in identical conditions, data highlights significant qualitative changes of sperm motility occurring from highly active sigmoid-like waveform in the control than a linear and weak flagellum movement in *HPX12* silenced mosquitoes^63^. In *Cx. quiquifaciatus*, in vitro studies suggest that the progression of sperm motility is calcium-dependent, where a very few sperms are motile in the absence of this cation^51^. Thus, evaluating the quality of sperm movement, especially in vivo is a critical factor to the fertility in animals including humans^64,65^ as well as insects/mosquitoes.

Further observation of increased expression of ROS generating transcripts, and altered MRO morphology in the *HPX12* knockdown mosquito’s testis, together with support the idea that *HPX12* may help to prevent the damage of sperms in adult male mosquitoes. Limited studies in the honey bee, *Apis mellifera* MRO, an increased expression of AOS enzyme transcripts such as catalase, glutathione-S-transferase (GST) and superoxide dismutase (SOD) have been suggested to contribute to sperm storage in the mature male reproductive organs^57^. However, the mechanism is relatively not well known in any male insect. In vertebrates, it is well evident that high susceptibility to reactive oxygen species (ROS), sperm production, and maintenance in the gonad is critical to the male reproductive system^66–68^, but this knowledge is lacking in mosquitoes.

While small amounts of ROS are required for normal sperm functioning, disproportionate levels can negatively impact the quality of spermatozoa and impair their overall fertilizing capacity^69^. Our observation of increased fluorescent intensity with a decrease in the sperm viability and motility in the unmated *HPX12* knockdown male mosquitoes supports the hypothesis that heme peroxidase enzyme *HPX12* plays an important role in the protection and storage of healthy sperms. However, we cannot exclude a contribution from other HPX members, especially HPX10 and HPX2 which also show a relatively higher expression in the virgin MRO (Supplemental Fig. 3d). Similarly, the importance of other non-HPX members in the regulation of stress physiology of male reproductive organs is unclear, and future studies targeting gene-specific strategies could help to address these questions.

Parallel to a significant reduction (50%) in the egg-laying, a remarkable observation of ~ 37% reduction in larval hatching from the laid eggs, further correlates that *HPX12* may serve as one of the important cellular factor in the maintenance of sperm quality and fertility of the mosquito *An. stephensi.* Also, a notable downregulation of tested Acps such as *ASTEI02706*, *ASTEI04746*, and *ASTEI10266*, appears to be responsible for the observed phenotype, likely by a decrement in semen quality. Although the exact mechanism of reduced egg-laying by gravid females is yet to be clarified, our data indirectly suggest that loss in *HPX12* expression may likely modify the property of the final ejaculate/seminal fluid, by altering the Acps expression, and/or quantitative loss in the sperm numbers.

Alternatively, we also interpret that an abundant transcript expression of *HPX12* in the testis may result in the synthesis and distribution of enzymes within the MRO sub-components including MAG, seminal vesicle, and testis. This strategy may help in maintaining optimal physiological homeostasis of the whole organ, not only for the protection and storage of healthy sperm in the testis, also for their viability/motility during the transfer of ejaculate to the female. Our data suggest that this is accomplished by scavenging harmful reactive oxiygen species (ROS). A recent, proteomic study in *Aedes aegypti* identifies that seminal fluid contains many proteins and enzymes including peroxiredoxin1 *(AAEL01940)*, catalase *(AAEL013407)*, and peroxidases *(AAEL013171)*, however, their functions has not been fully established^70^. Mammalian peroxiredoxin (*PRDX6*), which acts through its peroxidase and calcium-independent phospholipase A2 activities, plays an important protective role in the testicular physiology for the maintenance of sperm viability and motility^71,72^. Comparative conserved domain analysis of *Aedes aegypti* peroxidase (*AAEL013171*), and *An. stephensi* heme peroxidase (*AsHPX12*), confers that both species encode similar peroxinectin like *An_peroxidase* domain-containing proteins, but share only ~ 31% identity at amino acid level (Supplemental Fig. 9). *Aedes* peroxidase function is yet to unravel, here, we propose that *HPX12* supposedly has a significant impact on the male mosquito’s MRO physiology of *An. stephensi*.

In *An. gambiae* RNAi of Heat Shock Factors (HSFs) of the ejaculate further reduce at least 50% seminal fluid proteins (SFPs) expression but does not interfere with the male mosquito’s ability to mate^24^. In *An. gambiae*, Gabrieli et al.^43^ have shown that male transferred 20E hormone influences post mating behavioral resposes such as refractoriness to remating. Further studies highlighted that increased HPX15 expression in the female spermateca via male transferred 20E, seems to help in the protection of stored sperm^33^. Thus finding of two putative 20E binding sites in the *HPX12* gene, as predicted by JASPER software (Supplementary Table 3), indicates that the 20E hormone may also influence the regulation of *HPX12* expression, though experimental verification is required to validate the proposition. In summary, we propose that *HPX12* could serve as a possible molecular target to reduce the *Anopheles* male mosquito’s fertility in the field populations^6^.

## Supplementary Information

Supplementary Information 1.

Supplementary Video 1.

Supplementary Video 2.

Supplementary Video 3.

Supplementary Video 4.

Supplementary Video 5.

Supplementary Video 6.

Supplementary Video 7.

Supplementary Video 8.

Supplementary Video 9.

Supplementary Video 10.

## Data Availability

Uploaded as supplemental data.
