# Supplementary material for: A testis-expressing heme peroxidase HPX12 regulates male fertility in the mosquito Anopheles stephensi
Source: Sci Rep. 2022 Feb 16;12:2597. doi: 10.1038/s41598-022-06531-x (PMC8850455; doi:10.1038/s41598-022-06531-x)
Supplement: Supplementary file 1 — Supplementary Information 1. [file 41598_2022_6531_MOESM1_ESM.pdf]

Supplemental Data Sheet

A testis-expressing heme peroxidase HPX12 regulates male fertility in the mosquito *Anopheles stephensi*

Seena Kumari[1], Charu Chauhan[1], Jyoti Rani[1], Tanwee Das De[1], Sanjay Tevatiya[1], Punita Sharma[1], Kailash C Pandey[1], Veena Pande[2], Rajnikant Dixit[1]*

1. Laboratory of Host-Parasite Interaction Studies, ICMR-National Institute of Malaria Research, Dwarka, New Delhi-110077, India
2. Department of Biotechnology, Kumaun University, Nainital, Uttarakhand, India

***Correspondence:** Rajnikant Dixit**; Email:** dixitrk@mrcindia.org

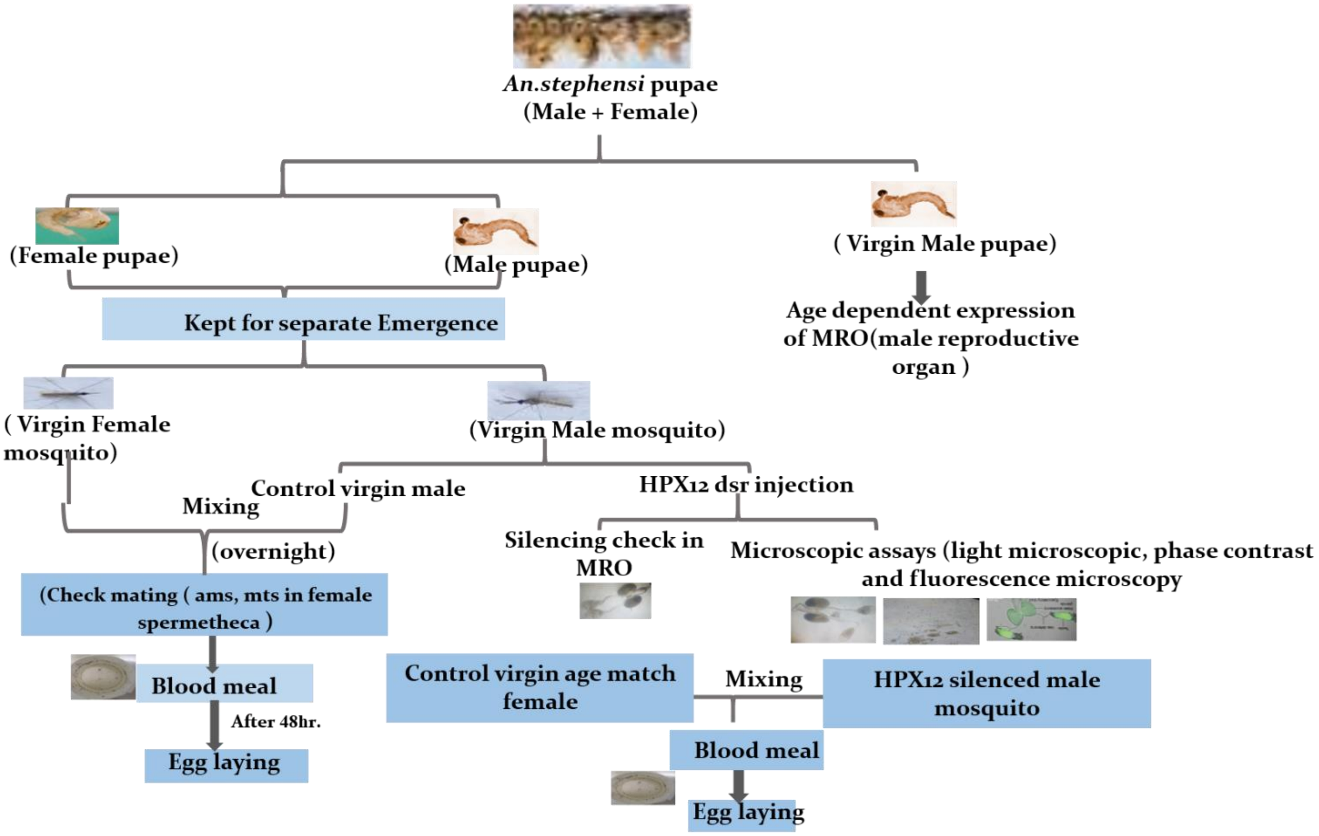

Supplementary Fig.1 Technical designing and experimental workflow

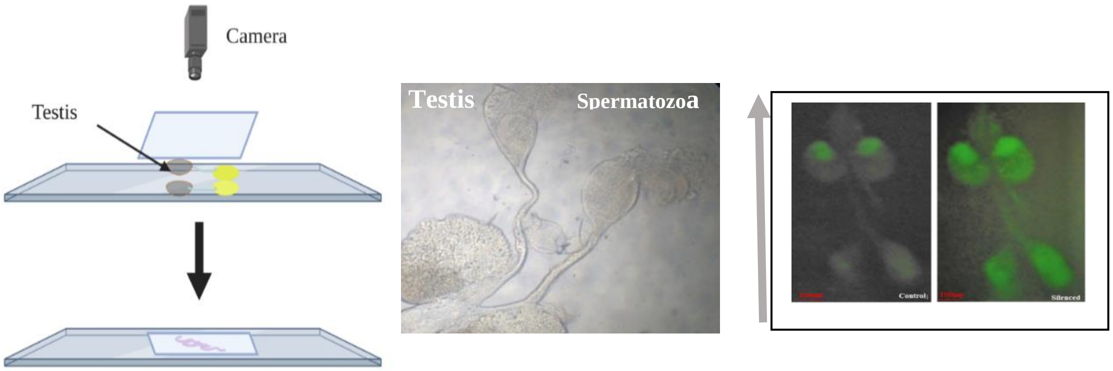

Supplementary Fig. 2 Sperm morphology and motility and ROS assay

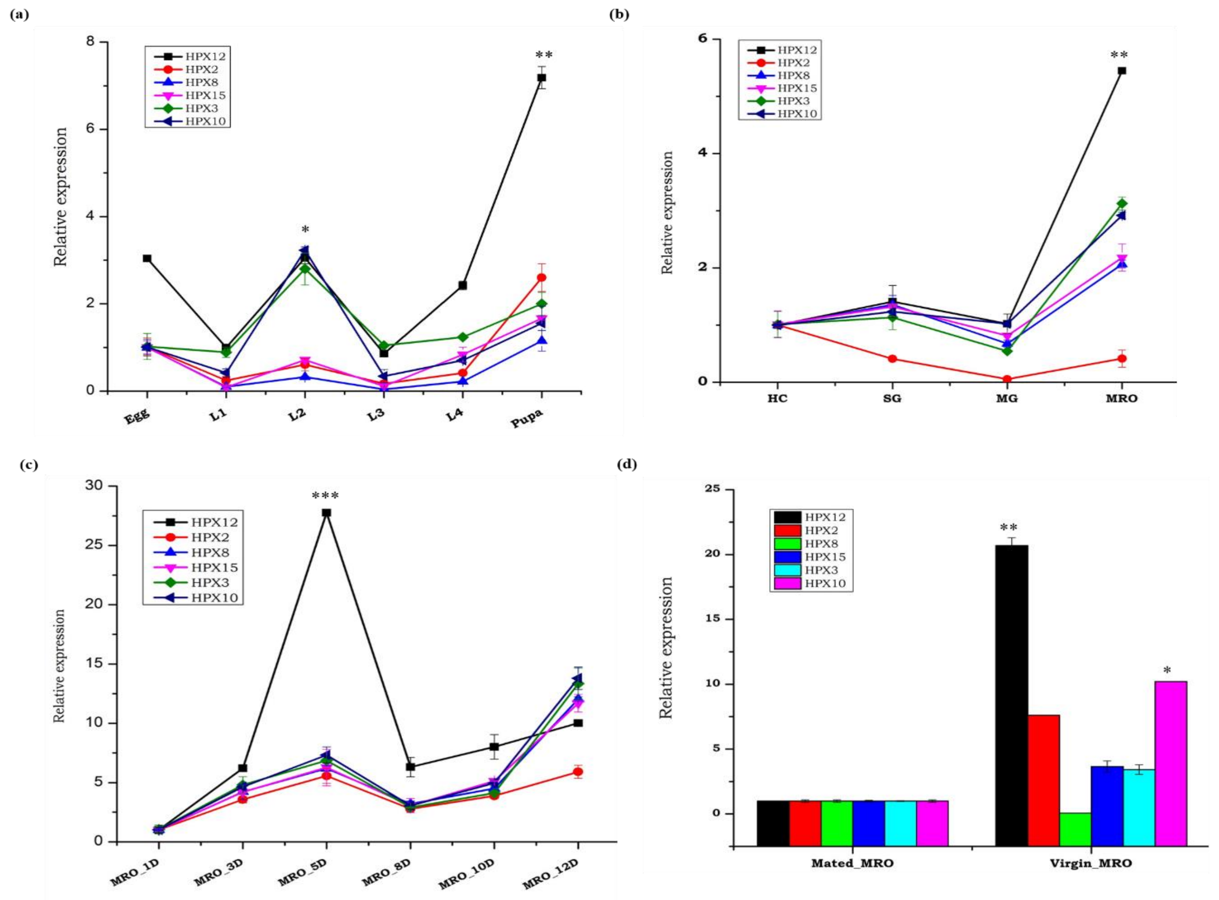

Supplementary Fig.3 Transcriptional profiling of the heme peroxidase family protein transcripts (hpx) in mosquito *An. stephensi* **(a)** Heme peroxidases (HPX12,10,15,3,8 and 2) expression during embryonic development: L1- larval instar one, L2- larval instar 2, L3-larval instar 3 and L4-larval instar forth, pupa ($p<0.0001$) (n= 3,N10);**(b)** Tissues specific expression kinetics of HPX family in male mosquito tissues SG: Salivary glands; MG: Midgut; HC: Hemocytes, MRO; male reproductive organs ($p<0.00045$); **(c)** Age-dependent expression of HPX family in virgin male mosquito reproductive organ (MRO) i.e. 1D(day),3D,5D ($p<0.0008564$),8D and 10D,12 days; **(d)** Mating-induced changes in hpx family in male reproductive organ Vir vs. Mated ($p<0.00324$). Three independent biological replicates (n=30, N3) were considered for statistical analysis viz: *$p<0.05$; **$p<0.005$ and ***$p<0.0005$ using un paired Student's *t-test*.(*n*=represents the number of mosquito pooled for sample collection; *N*= number of replicates)

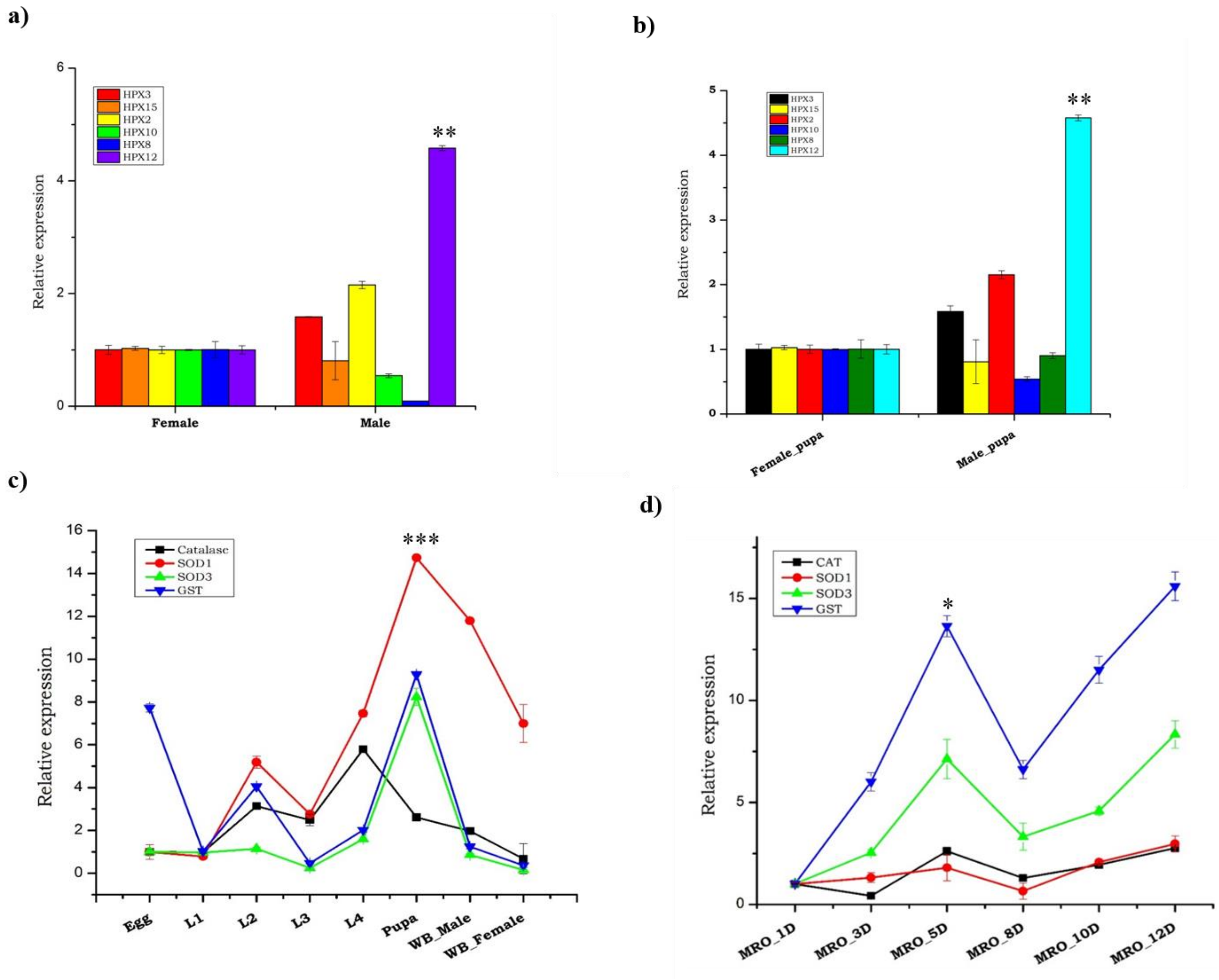

Supplementary Fig.4 Transcriptional profiling of the antioxidant related (AOS) genes transcripts (hpx) in mosquito *An. stephensi* **a)** Heme peroxidases (Heme peroxidase 12,10,15,3,8 and 2; *HPX12/p<0.000388) expressions in male and female whole body (n=3, N1); **b)** Transcriptional profiling of the HPX family in a male and female pupal stage: HPX12 (p<0.000144954), HPX2, HPX8, HPX10, HPX15, and HPX3. The female pupal stage was considered as controls for each test sample for respective genes**. c)** AOS genes i.e. SOD1(Superoxide dismutase 1), SOD3 (Superoxide dismutase3),GST (Glutathione-s-transferase) and catalase proliferation in aquatic stage i.e. L1- larval instar one, L2- larval instar 2, L3-larval instar 3 and L4-larval instar forth, pupa (p<0.0001, n= 3, N10), L1 sample was considered as controls for each test sample for respective genes; **d)** Age-dependent expression of AOS family in virgin male mosquito reproductive organ (MRO) i.e. 1D (day), 3D, 5D (p<0.008564), 8D and 10D, 12 days. female whole body sample was considered as controls for each test sample for respective genes. Three independent biological replicates (n=30, N3) were considered for statistical analysis viz: *p<0.05; **p<0.005 and ***p<0.0005 using unpaired Student's *t-test*. (*n*=represents the number of mosquitoes pooled for sample collection; *N*= number of replicates) and final *p-values* were adjusted using Benjamini & Hochberg test**.**

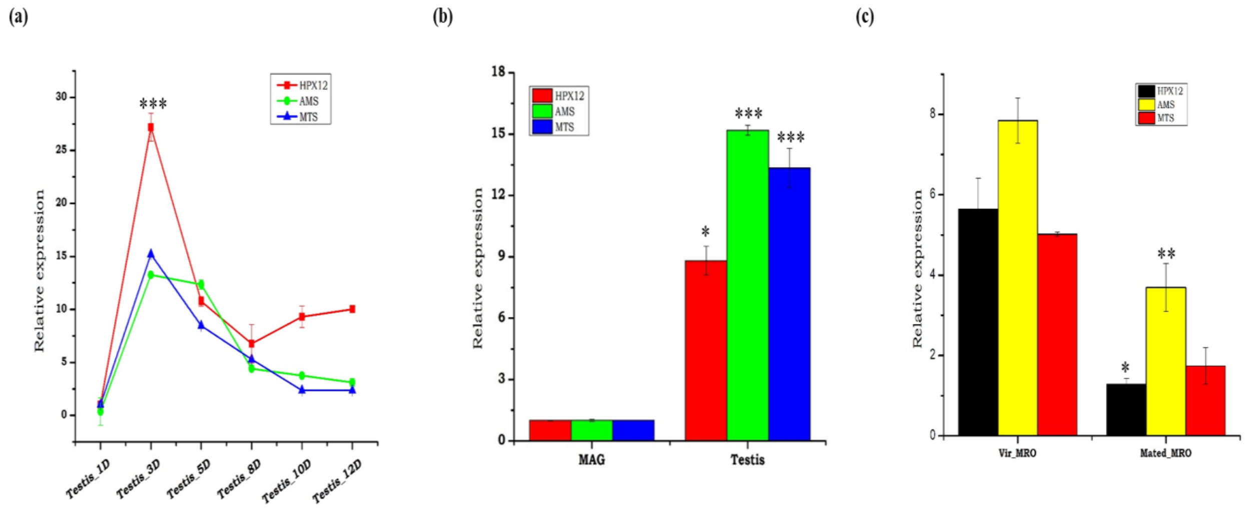

Supplementary Fig. 5 Transcriptional profiling of the HPX12 and sperm-specific transcripts (AMS, MTS) in mosquito *An. stephensi* **a)** Age-dependent co-expression of hpx12 and ams, mts in virgin male testes, 1D,3D,5D,8D, 10D, and 12D, and one-day testis sample was considered as controls for each test sample for respective genes. b) Transcriptional profiling of hpx12 (p<0.01504) and ams(p<0.000894), mts (5.57E-05) in male reproductive tissues (testis, mag) and MAG: (male accessory gland) sample was considered as controls for each test sample for respective genes; c) Mating-induced changes in hpx12 (p< 0.01010), mts (p<0.002276) and ams (p<0.035324) in male testis Virgin vs. Mated. Three independent biological replicates (n=3, N30) were considered for statistical analysis viz. *p<0.05; **p<0.005 and ***p<0.0005 using Student's *t-test* and final p-values were adjusted using Benjamini & Hochberg test**.**

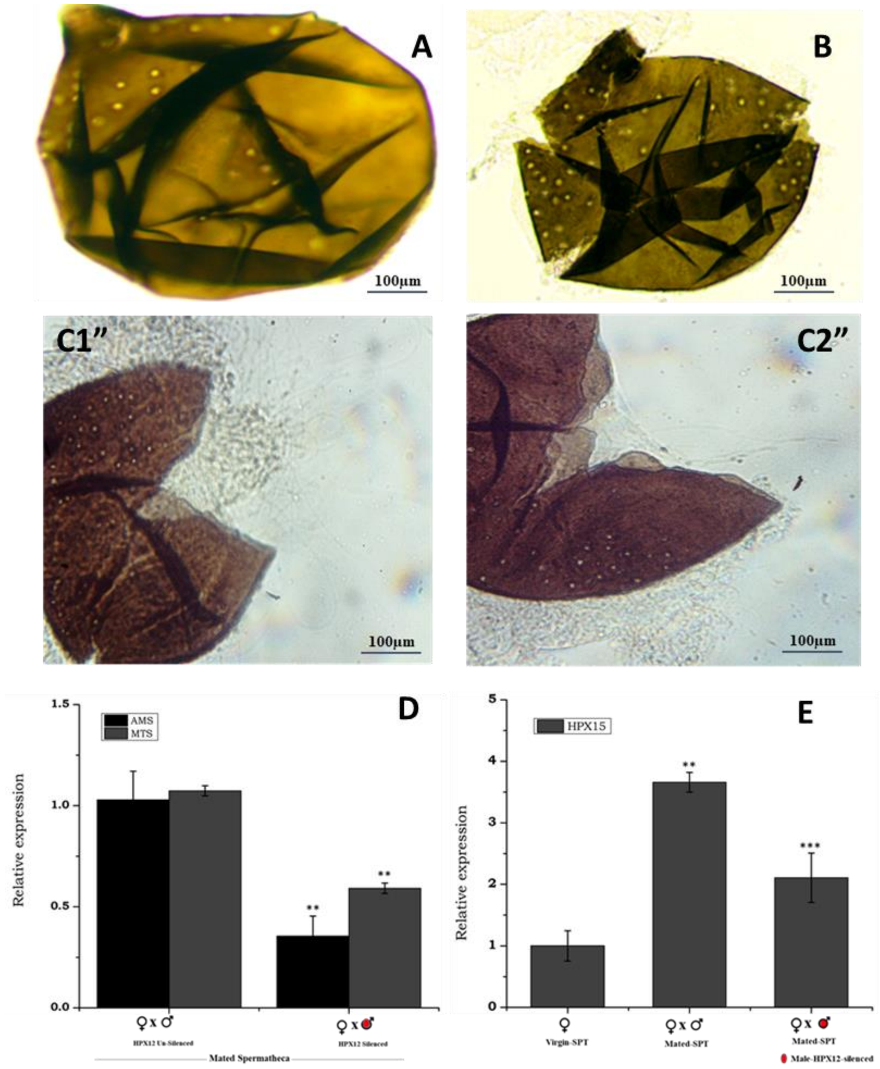

Supplementary Fig. 6: Morphological and molecular analysis of spermatheca: (A-B):-Microscopic view of unmated i.e. virgin (A) and mated (B) female mosquito spermatheca; **(C1”-C2”):-** Mating with HPX12 silenced male mosquito may cause lesser sperm receipt than control mosquitoes as shown by comparative microscopic view of ruptured spermatheca of female mosquitoes mated with naïve control or HPX12 silenced male mosquitoes, respectively; **(D)**: as well as by comparative quantitative transcriptional profiling of sperm-specific genes (AMS & MTS) in the spermatheca of female mosquitoes mated with naïve control or HPX12 silenced male mosquitoes, respectively; **(E):** Comparative quantitative transcriptional profiling of HPX15 in the female mosquitoes spermatheca of virgin, mated with naïve control or HPX12 silenced male mosquitoes, respectively.

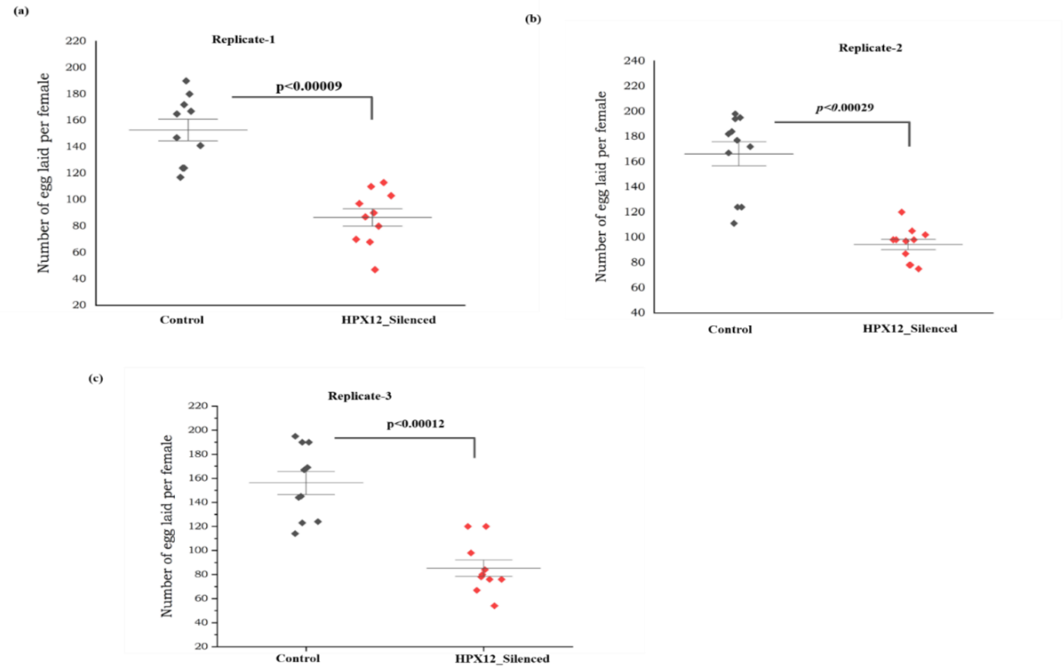

Supplementary Fig. 7: Mating with HPX12 silenced male mosquitoes reduces the egg-laying capacity of adult female mosquitoes: laid egg number count by females in control (injected with ds*LacZ)* and HPX12 silenced mosquitoes in three independent replicates. Females were allowed to lay eggs between days 4 and 5. (n=15–25 females/trial) and each replicates p-value p<0.00009,p<0.00029, and p<0.00012 (Mann- Whitney *U* test).

(A) ***ASTEI02706***

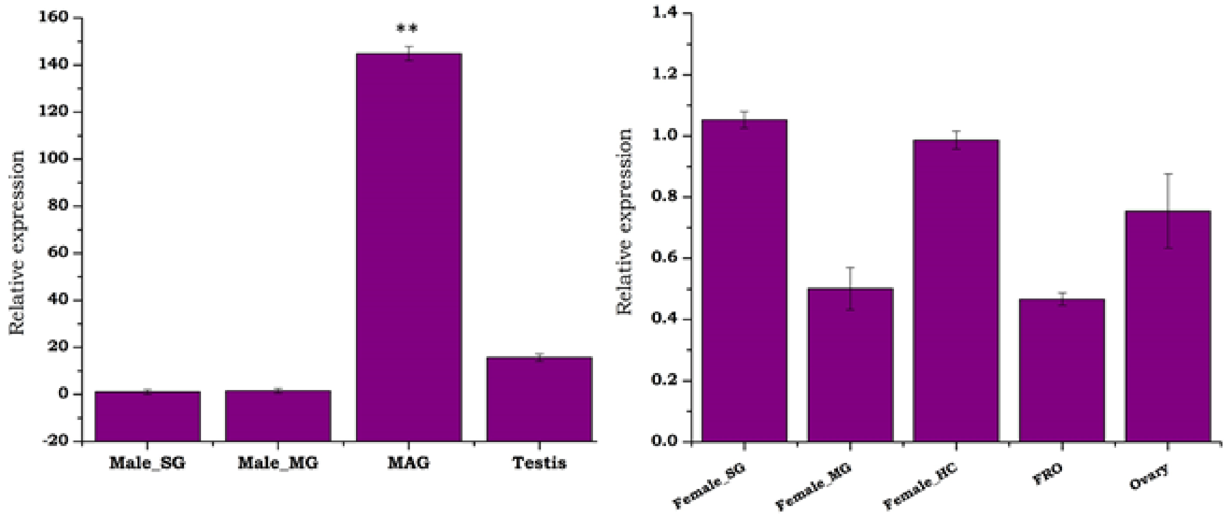

(B) ***ASTEI10266***

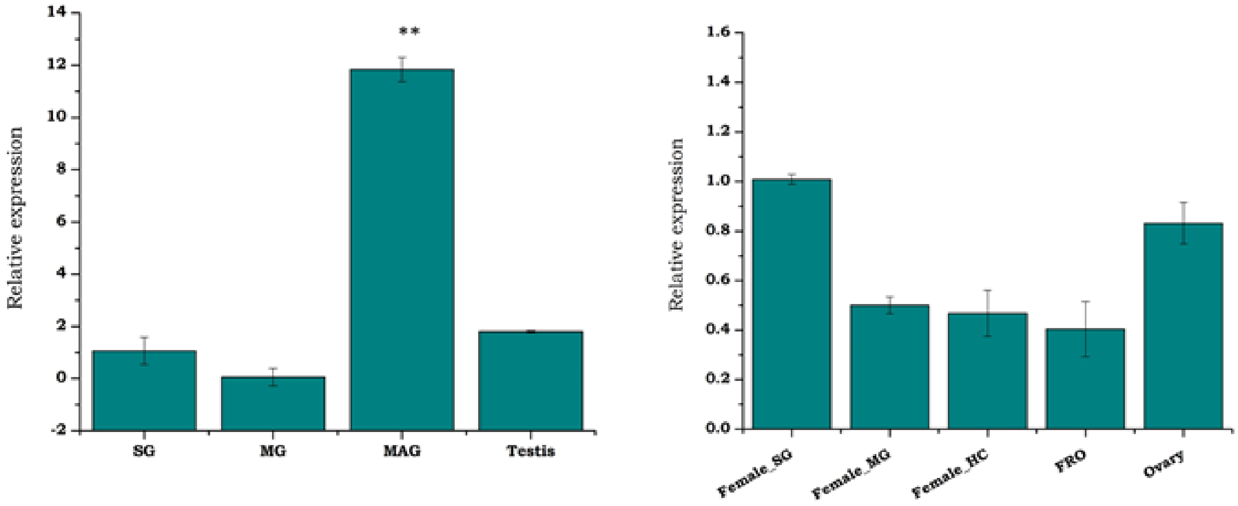

(C) ***ASTEI00744***

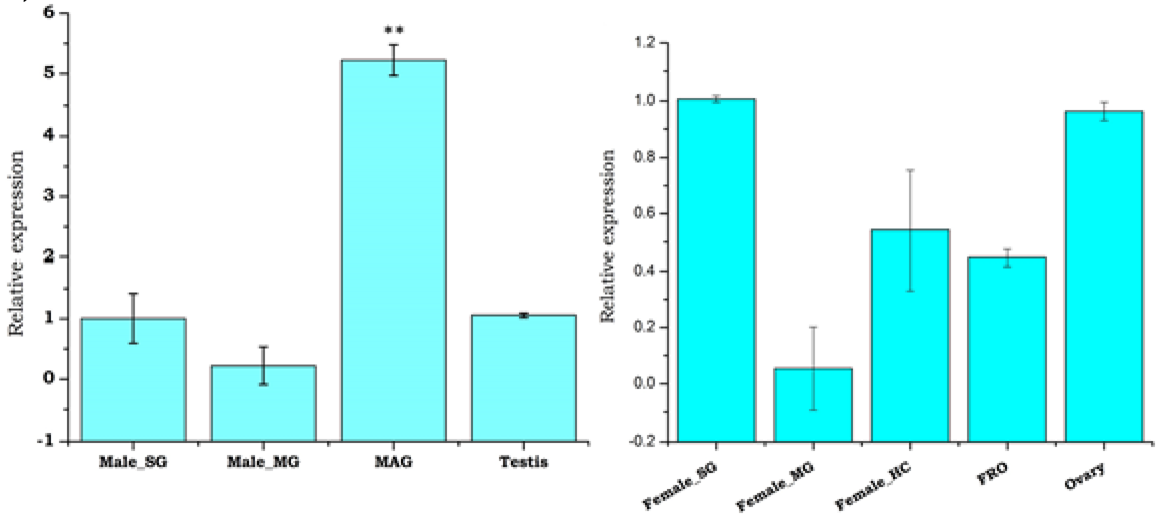

Supplementary Fig.8: Sex and tissue-specific ttranscriptional profiling of the putative accessory gland proteins (ACPs) encoding transcripts in mosquito *An. stephensi;* Three independent biological replicates (n=3, N30) were considered for statistical analysis viz. *p<0.05; **p<0.005 and ***p<0.0005 using Student's *t-test*, and final p-values were adjusted using Benjamini & Hochberg test.

(A)

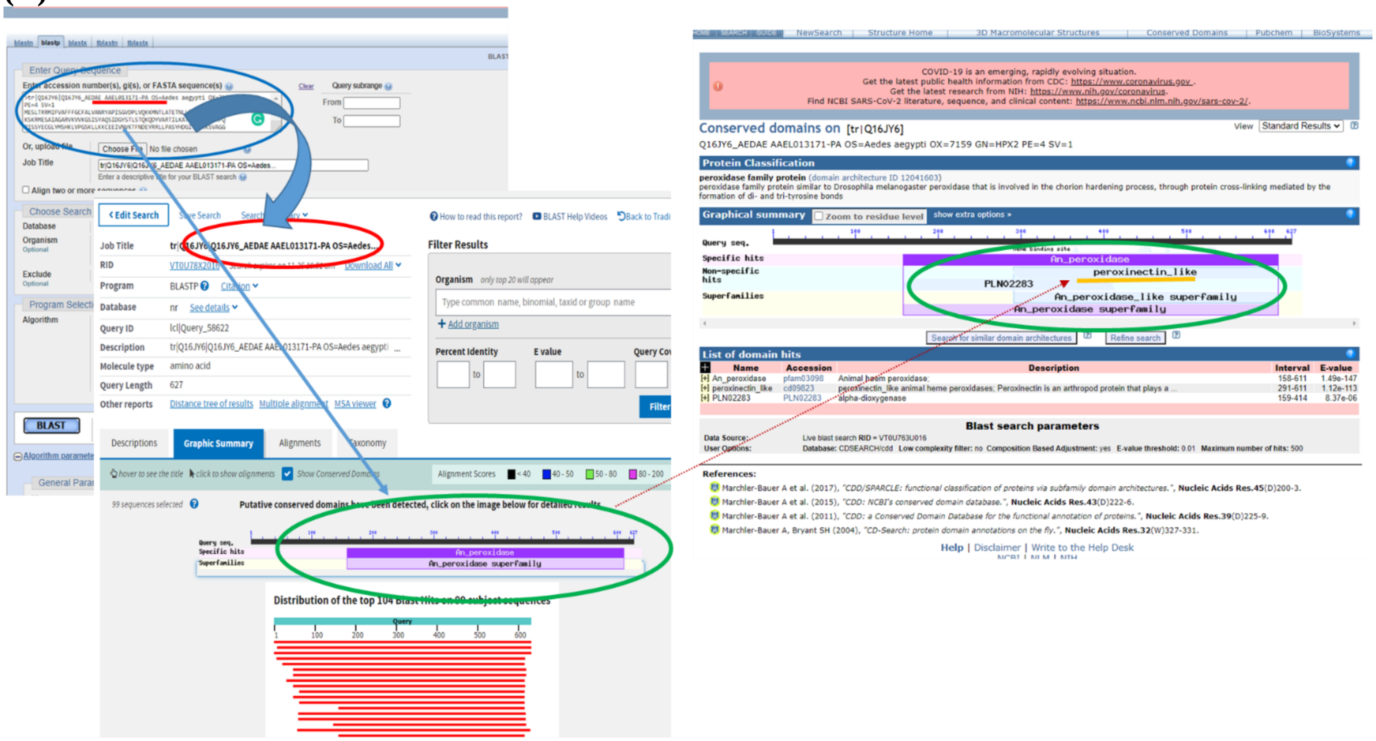

Job Title
Program
Database
Query ID
Description
Molecule type
Query Length
Other reports
Descriptions
Graphic Summary
Alignments
Taxonomy
Filter Results
Conserved domains on [tr|Q16JY6]
Protein Classification
Graphical summary
List of domain hits
Blast search parameters
References:

(B)

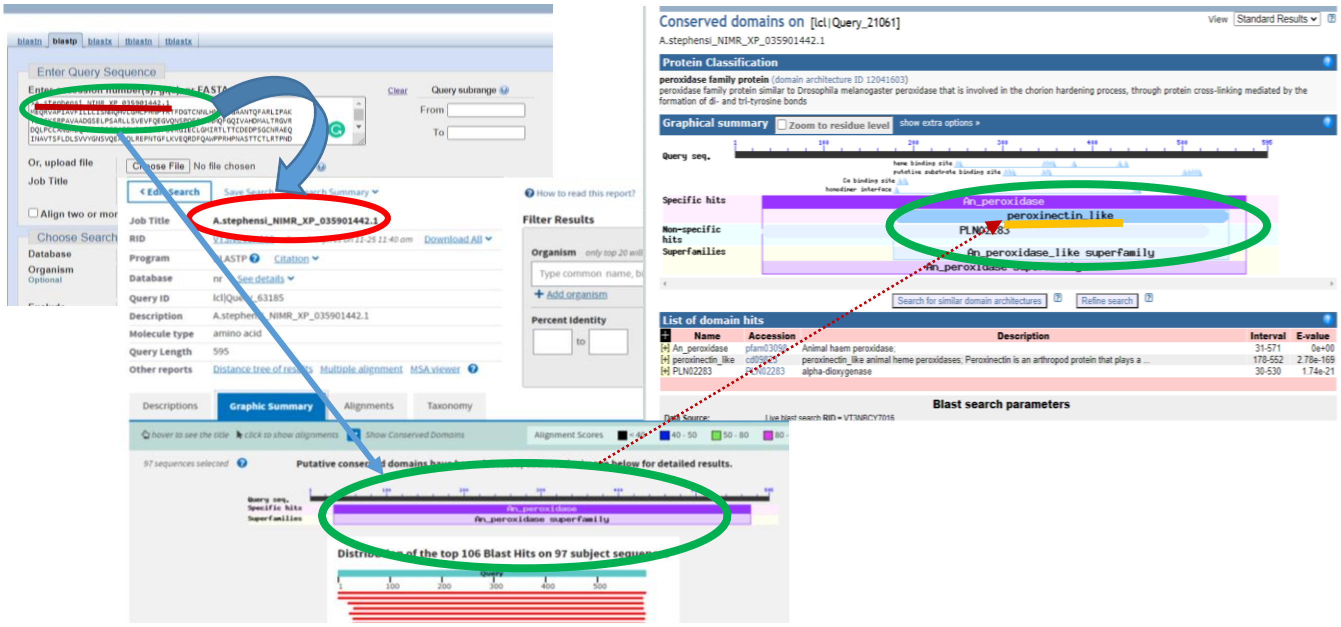

Enter Query Sequence
Or, upload file
Job Title
Choose Search
A.stephensi_NIMR_XP_035901442.1
Filter Results
Descriptions
Graphic Summary
Alignments
Taxonomy
Conserved domains on [lcl|Query_21061]
A.stephensi_NIMR_XP_035901442.1
Protein Classification
Graphical summary
List of domain hits
Blast search parameters

(C)

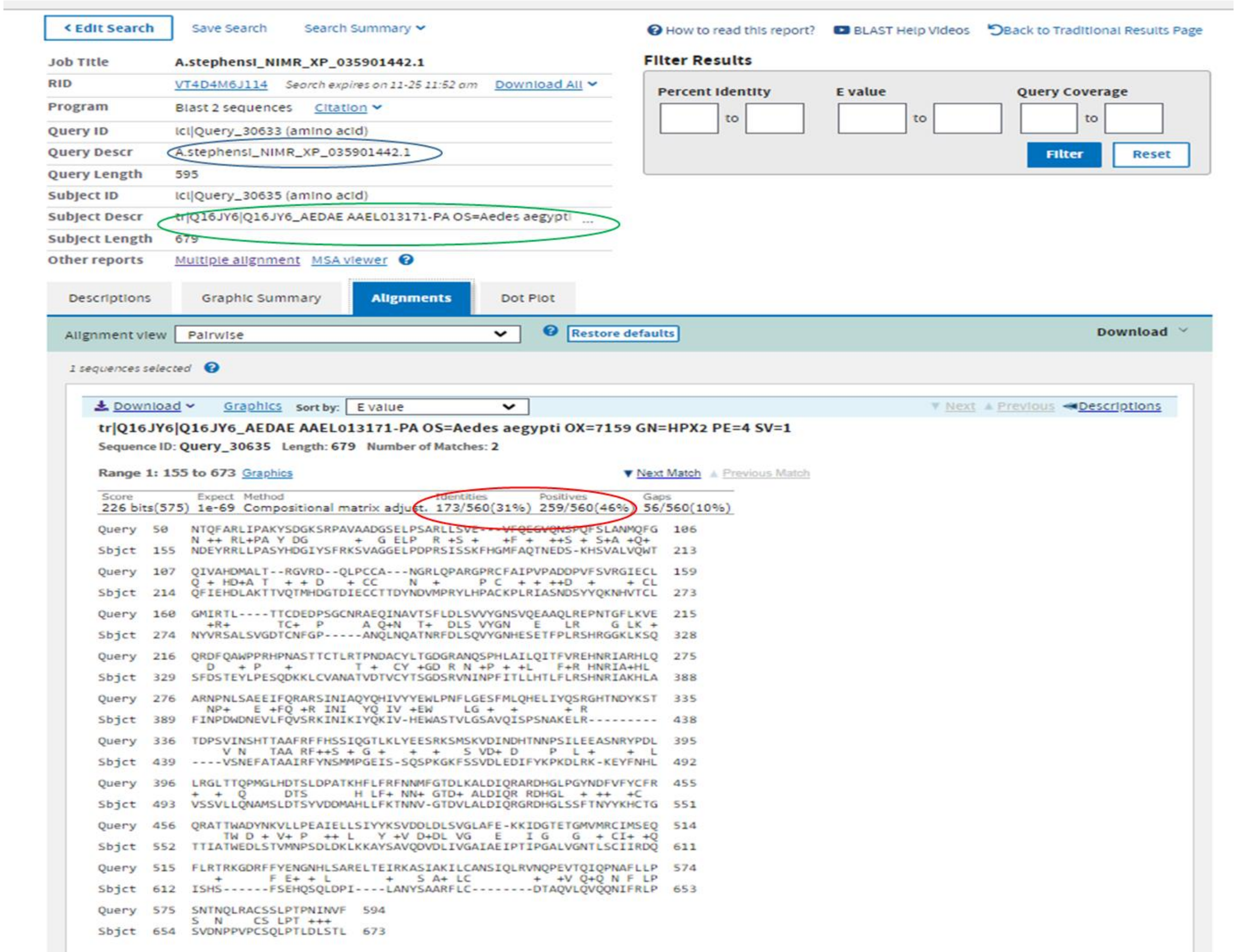

Supplementary Fig.9: Comparative in silico analysis of putative peroxidase (AAEL013171) identified from MRO of *Aedes aegypti* **[A]** *(*Degner et al., 2019, Molecular & Cellular Proteomics 18, S6 –S22) & HPX12 from *A. stephensi*_NIMR_XP_035901442.1 **[B]**; characterized in the current investigation, showing both transcript encode putative peroxidase carrying *An_peroxidase*/ PEROXINECTIN like domain containing protein, and share 31% identity **[C]**, and thus we propose that similar to *An. stephensi*, *Aedes* peroxidase may have similar function of testis physiological homeostasis maintenance, and need to validate functionally.

Supplementary Table.1 Primer list

S.No.	Gene Name	Primer Sequence
1	HPX2	Fw: CACGAAGCTAAAAATTGTCC<br>Rev: AAGAGATGCTCCAGATCGTA
2	HPX3	Fw: AGTTCTTCACGGTTCATCAC<br>Rev: GATCTTCTCCAGGTGTTTTG
3	HPX8	Fw: AAGCTGTACCAAGAAGCTC<br>Rev: GCTCCTGATACTTCGTTAAA
4	HPX10	Fw: AAGAAGGTTGACGGTACAGA<br>Rev: CAACGTTAGCAGTCGTATCA
5	HPX15	Fw: CTATTGTCGCAACAGTACGA<br>Rev: CGGTAAACTGTCCATCATTT
6	ASTEI2706	Fw: 5' TACACGCTAGTGGTTTTGC 3'<br>Rev: : 5’ AGCAAGATGTGGAAGGAATA 3’
7	HPX12	Fw: GAACAGTGCCACCGATACCT<br>Rev: CCGAGATAATAGGGCAACCA
8	ASTE00744	Fw: TCATAATGTGGTGTACAAGC<br>Rev: ACCTAATCTCTATCGGTGTG
9	Catalase	Fw: 5' CCCAACTATTTCCCGAAC 3'<br>Rev: 5’CAGGTGTCCCACAATGTT3'
10	GST	Fw: 5' AACGGGTCGTCGATTACT 3’<br>Rev: 5' AGGTCGAACTGGAATGCT 3’
11	DsrLacz	Fw: 5' TAATACGACTCACTATAGGGGAGTCAGTGAGCGAGGAAG 3'<br>Rev: 5’TAATACGACTCACTATAGGGTATCCGCTCACAATTCCACA 3’
12	DsrHPX12	Fw: 5’ TAATACGACTCACTATAGGGTTCTGGTGTTTGCCATCGTA 3’<br>Rev: 5’ TAATACGACTCACTATAGGGCAGGATGTTCTGCTCGTTGA 3’
13	SOD1	Fw: 5' TGGGAGCACGCTTACTAT 3’<br>Rev: 5' GCTGTCGACTTTTGGCTA 3’
14	SOD3	Fw: 5' GATCGTCCGTCGCTATTA 3’<br>Rev: 5' CCTGCAAGCGTTATCTTC **3’**

15	ASTE110266	Fw: 5' GTTTTACGGAGTAACCAAGA 3’<br>Rev: 5' TACGGCATAGTTACAAACTG 3’
16	Sulfhydryl oxidase	Fw: 5' TTTTACAACTCGTACTGTGG 3’<br>Rev: 5' TCACTGGTTCTCCTATCTTC 3’
17	Actin	Fw: 5’ TGCGTGACATCAAGGAGAAG 3’<br>Rev: 5’GATTCCATACCCAGGAACGA 3’
18	AMS	Fw: 5’ AATCGTGAAGTGATTGATCT 3 ‘<br>Rev 5’ AGAGGCACGAGTAGTGTTAC 3’
19	MTS	Fw: 5’ ATGTACGAGGTGTTCAACTT 3 ‘<br>Rev 5’ GACAATCTTGAAGAATCCAC
20	Plugin	Fw: 5’ ACAATTTGCAAAACTTTCTC<br>Rev 5’ GTAGCGAGTTGTTGTTGTTA

Supplementary Table 2

	Putative Acp	Accession ID (*An. gambiae)*	*An. stephensi* (Transcript ID)	Percent Identity to *An. gambiae*	Conserved domain	Expression	Remarks
1.	Serine protease like superfamily (ACP62F)	AGAP006587-RA	ASTEI2706-RA	31.7%	TIL Domain	MAG	Protease Inhibitor like activity
2.	P-450	AGAP002429-RA	ASTE00744-RA	88.35%	Cytochrome P-450 Domain	MAG	Likely involved to catalyze one of the ecdysteroid biosynthesis oxidative steps
3.	Venom allergen	AGAP006418-RA	ASTE110266-RA	66.41%	(CAP Domain)	MAG	Venom allergen 5/antigen 5, a cysteine-rich secretory protein superfamily. High-level expression of this gene in the MAGs and ejaculatory ducts in Insect *Bactrocera dorsalis*, and in mammalian testes, where it is involved in spermatogenesis (
4.	TGM	ACZ65013.1	ASTE010111-RA	70.82%	TGC (Transglut-N-Superfamily)	FRO	Transglutaminase-Mediated semen Coagulation Controls Sperm Storage in the Malaria Mosquito
5	Staufen	AGAP007478-RA	ASTEI01398-RA	67%	DSRM	MAG/FRO	RNA-binding activity

Supplementary Table 3. JASPER result analysis:

Matrix ID	Name	Score	Relative score	Predicted sequence
MA0026.1	Eip74EF	10.7555	1.000000014	CCGGAAG
MA0534.1	EcR::usp	7.53989	0.831347329	CAGGATATTCAACTC

Videos: S1-S5

Comparative observation of Control & HPX12 silenced male mosquito's Spermatozoa flagella motility videos

Video-S1: Control-Replicate-1

Video-S1: HPX12_Silenced-Replicate-1

Video-S2: Control-Replicate-2

Video-S2: HPX12_Silenced-Replicate-2

Video-S3: Control-Replicate-3

Video-S3: HPX12_Silenced-Replicate-3

Video-S4: Control-Replicate-4

Video-S4: HPX12_Silenced-Replicate-4

Video-S5: Control-Replicate-5

Video-S5: HPX12_Silenced-Replicate-5
